# What explains the fall in child stunting in Sub-Saharan Africa?

**DOI:** 10.1016/j.ssmph.2019.100384

**Published:** 2019-05-13

**Authors:** Leander R. Buisman, Ellen Van de Poel, Owen O'Donnell, Eddy K.A. van Doorslaer

**Affiliations:** a Erasmus School of Health Policy & Management, Erasmus University Rotterdam, Rotterdam, Netherlands; b Department of Applied Economics, Erasmus School of Economics, Erasmus University Rotterdam, Rotterdam, Netherlands

**Keywords:** Nutrition, Stunting, Decomposition, Sub-Saharan Africa

## Abstract

There have been steep falls in rates of child stunting in much of Sub-Saharan Africa (SSA). Using Demographic and Health Survey data, we document significant reductions in stunting in seven SSA countries in the period 2005–2014. For each country, we distinguish potential determinants that move in a direction consistent with having contributed to the reduction in stunting from those that do not. We then decompose the change in stunting and in proximal determinants into a part that can be explained by changes in distal determinants and a residual part that captures the impact of unmeasured factors, such as vertical nutrition programs. We show that increases in coverage of child immunization, deworming medication and maternal iron supplementation often coincide with a fall in stunting. The magnitudes and directions of changes in two other proximal determinants -- age-appropriate feeding and diarrhea prevalence -- suggest that these have not been strong contributors to the fall in stunting. Utilization of maternity care emerges from the decomposition analysis as the most important distal determinant associated with reduced stunting, and also with increased coverage of iron supplementation, and, to a lesser extent, with child immunization and deworming medication. This circumstantial evidence is strong enough to warrant more detailed investigation of the extent to which maternity care is an effective channel through which to target further attacks on the blight of undernourished children.

## Highlights

• The rate of children (<2 years old) who are stunted fell in many Sub-Saharan African countries in recent decades.
• This reduction was associated with improved access to maternity care.
• The improved access to maternity care may proxy for improvements in good quality preventive care.
• The reduction in stunting was not associated with improved feeding practices or reduced incidence of diseases.

## 1 Introduction

Globally, the average rate of stunting fell between 2005 and 2015 (UNICEF-WHO-World Bank, 2017). Economic growth, improved socioeconomic conditions, increased access to healthcare, nutrition and other vertical child health programs, and behavioral changes, such as increased breastfeeding and reduced fertility, may all have contributed to this welcome improvement in the wellbeing of children (Headey, Hoddinott, Ali, Teshaye, & Dereje, 2015; Smith & Haddad, 2015). Much of Sub-Saharan Africa has experienced the improvement (Bredenkamp, Buisman, & Van de Poel, 2014; UNICEF-WHO-World Bank, 2017). Unfortunately, not all African children have benefited, however. Rates of stunting have remained stubbornly high, or have even risen, in Malawi, Mozambique and Sierra Leone (Bredenkamp et al., 2014; World Development Indicators, 2019). To avoid children in these countries being left too far behind, it is important to learn from the success stories about what contributes to improvements in nutritional status. This paper documents changes in stunting in nine Sub-Saharan African (SSA) countries. For the seven in which stunting fell significantly, it decomposes the reductions into changes in potential determinants, with the goal of distinguishing factors that plausibly could have contributed to these reductions from those that could not.

Identification of the causes of nutritional status is difficult. Certainly, it cannot be done by regressing nutritional outcomes on proximal determinants (Hien & Hoa, 2009; Larrea & Kawachi, 2005; Marquis, Habicht, Lanata, Black, & Rasmussen, 1997). First, the timing of some measures rules out a causal effect between them: previous day feeding cannot impact on a child's measured height. Second, there is reverse causality: undernutrition causes illness. Third, nutritional inputs, such as feeding, are behaviorally determined and likely correlated with unobserved determinants of nutritional status, such as hygiene and health knowledge. Recognizing these problems, attempts to explain trends in stunting have mostly paid attention to the contribution of more distal determinants (Wagstaff, van Doorslaer, & Watanabe, 2003; Van de Poel, Hosseinpoor, Jehu-Appiah, Vega, & Speybroeck, 2007; Headey, 2014; Headey & Hoddinott, 2014; Headey et al., 2015; Chirande et al., 2015; Salvucci, 2016). A limitation of this approach is that it loses sight of the immediate, biological mechanisms through which inputs and stresses impact on nutritional status. We avoid this limitation by examining trends in nutritional status and its proximate determinants without attempting to estimate the causal relationships between them, and by assessing the extent to which each can be explained by more distal determinants of nutrition. The objective is to obtain circumstantial evidence on what may have contributed to the reductions in stunting rather than aiming for definitive evidence on the causes.

We use the UNICEF framework (1990), and its adaptation in the Lancet Maternal and Child Nutrition Series (2013), to distinguish proximal from distal determinants of nutritional status. We separate proximate determinants that move in a direction consistent with having contributed to the reduction in stunting from those that either display no trend or move in a direction that would impact negatively on nutritional status. We then decompose the change in stunting, as well as the mean height-for-age z-score (HAZ) and mean values of the proximal determinants that plausibly could have contributed to the reduction in stunting, into a part that can be explained by changes in distal determinants and a part that is left unexplained. This residual component captures the impact of nutritional inputs, vertical programs and other determinants of nutritional outcomes that are not measured.

We show that in at least 4 of the 7 countries in which stunting fell significantly, the decline is coincident with increased coverage of child immunization, deworming medication and maternal iron supplementation. Two other proximal determinants -- age-appropriate feeding and diarrhea prevalence – do not appear to have been strong contributors to the observed fall in stunting. They have improved insufficiently, or not at all. From the decomposition analysis, maternity care emerges as the most important distal determinant associated with reduced stunting. Increased utilization of maternity care is also associated with increased uptake of preventive services, particularly maternal iron supplementation and, to some extent, child immunization and deworming medication.

Unlike our within country analysis (for a number of countries), Headey (2013) conducts cross-country comparisons and also finds that increased access to maternity care explains a substantial part of the variation in the nutritional status of children. In addition, he finds wealth (asset ownership), maternal education and lower fertility to be strong predictors of improved nutritional status. Through longer birth intervals, we also find an important role for lower fertility, and we find a modest contribution from maternal (and paternal) education. Another cross-country study by Smith and Haddad (2015) identifies economic growth as an important distant driver of reductions in stunting. In contrast to our analysis, this paper also finds improved drinking water and sanitation to be important predictors. Country-specific analyses of Bangladesh (Headey et al., 2015), Ethiopia (Headey, 2014) and Nepal (Headey & Hoddinott, 2014) all find that wealth, maternity care, parental education and sanitation are significantly associated with improved nutritional status.

## 2 Material and methods

### 2.1 Data

We use Demographic Health Survey (DHS) data from nine Sub-Saharan African (SSA) countries to document changes in child stunting between 2005 and 2014. For each country, we use data from two survey waves 4–7 years apart: Ethiopia (2005, 2011), Ghana (2008, 2014), Kenya (2008, 2014), Liberia (2007, 2013), Namibia (2006, 2013), Niger (2006, 2012), Rwanda (2010, 2014), Sierra Leone (2008, 2013), and Zambia (2007, 2013). The country inclusion criteria were entirely based on availability of data. That is, data on child anthropometrics and selected determinants (Table 1) needed to be available from two DHS with the first survey occurring no earlier than 2005.^1^ All the DHS used are nationally-representative household surveys.

**Table 1:** Definitions of variables.

	Variable	Definition
***Outcomes***		
	Height-for-Age z-score	Height minus median height of child same age (months) and gender in reference population divided by standard deviation of height in reference population. Reference population is WHO, 2006 Child Growth Standards.
	Stunted	1 if Height-for-Age z-score < -2, 0 otherwise
***Proximal determinants***		
	Poor age-appropriate feeding	1 if poorly fed the previous day, 0 otherwise. Poorly fed: …. 0–5 months – option 1: not breastfed but complementary feeding and/or milk-based liquids; option 2) breastfed plus milk-based liquids and/or complementary feeding – but also allows water-based liquids to have been consumed. ….6–23 months – complementary feeding from 0 to 1/7 food groups
	Partial age-appropriate feeding	1 if partially appropriately fed the previous day, 0 otherwise. Partially appropriately fed: …. 0–5 months – breastfed plus water based liquids. …. 6–23 months –complementary feeding from 2 to 3/7 food groups
	Recommended age-appropriate feeding	1 if appropriately fed the previous day, 0 otherwise. Appropriately fed: …. 0–5 months – exclusively breastfed (breast milk only, but allows for oral rehydration salts). ….6–23 months – complementary feeding from ≥ 4/7 food groups
	Mother received iron supplements	1 if mother received iron tablets or syrup during pregnancy and took it for at least 1 day, 0 otherwise
	No symptoms of diarrhea	1 if child reported not to have had any symptoms of diarrhea in the past two weeks, 0 otherwise
	Full immunization	1 if child aged 12–23 months received BCG, measles and three doses of polio and DPT, either verified by card or by recall of mother in the first 2 life years, 0 otherwise. Polio given at time of birth does not count towards ‘full immunization’.
	Deworming medication	1 if child aged 12–23 months received deworming medication in past 6 months, 0 otherwise.
***Distal determinants***		
	Mother had 1-3 ANC visits	1 if birth preceded by 1–3 antenatal care (ANC) visits from any skilled personnel, 0 otherwise
	Mother had 4+ ANC visits	1 if birth preceded by at least 4 antenatal care (ANC) visits from any skilled personnel, 0 otherwise
	Delivered by skilled birth attendant	1 if birth attended by any skilled personnel, 0 otherwise
	Mother has any education	1 if mother had any education, 0 otherwise.
	Father has any education	1 if father/step-father had any education, 0 otherwise.
	Wealth index in top 60%	1 if household wealth index in top 60% of distribution, 0 otherwise.
	Surface water source (unimproved)	1 if surface water (unimproved) is source of drinking water, 0 otherwise
	Other unimproved water source (other than surface water)	1 if unimproved water other than surface water is source of drinking water, 0 otherwise.
	Improved water source	1 if improved water (per WHO guidelines) is source of drinking water, 0 otherwise.
	Open defecation (unimproved)	1 if no sanitation facilities (per WHO guidelines), 0 otherwise
	Other unimproved sanitation facility (other than open defecation)	1 if unimproved sanitation facilities other than open defecation, 0 otherwise.
	Improved sanitation facility	1 if improved sanitation facilities (per WHO guidelines), 0 otherwise.
	Have livestock	1 if household owns livestock, 0 otherwise
	Birth order	Birth order of child
	Birth interval >24 months	1 if child born more than 24 months after previous birth of mother, 0 otherwise
	Mother taller than 150 cm	1 if mother taller than 150 cm, 0 otherwise
	Mother's age at birth (in years)	Mother's age at child's birth in years
	Urban	1 if child lives in urban area, 0 otherwise
	Region	Household's region of residence. The regions are country-specific.
	Wet season	1 if interview conducted in wet season, 0 otherwise
	Child's age	Child's age in months grouped in 6-month categories: (1) 0–5 months, (2) 6–11 months, (3) 12–17 months, (4) 18–23 months.
	Child is male	1 if child is male, 0 otherwise

Unless otherwise noted, all children are under the age of 2, all mothers are aged 15 to 49 and had a birth in the past 2 years. Data on feeding, antenatal care and iron supplements only collected for the youngest child.

We focus on children aged less than 24 months to cover the 1000-day window from gestation when there is the potential for rapid physical and mental development and after which it is feared that any stunting that has manifested may be largely irreversible (Addo et al., 2015; Martorell, Melgar, Maluccio, Stein, & Rivera, 2010; Thurow, 2016). Above the age of two, improvements in proximal and distal determinants may be ineffective in fully recovering previous losses in potential height (Victora et al., 2008).^2^ Preventive programs that focus on nutrition and healthy growth from pregnancy to 24 months after birth are claimed to be most effective in securing health benefits that last throughout life (Bryce et al., 2008; WHO, 2008).

We examine three indicators of nutritional status – the mean height-for-age z-score (HAZ) and the proportions of children who are stunted and severely stunted. A child is stunted (severely) if it is two (three) standard deviations below the median height of a well-nourished child of the same age and gender using the World Health Organization Child Growth Standards (WHO, 2006). Height is measured at the time of the survey interview. HAZ is a measure of long term nutritional status that results from cumulative nutrition inputs and stresses from gestation to current age.

We distinguish between *proximal* and *distal* determinants of child nutritional status (Lancet Maternal & Child Nutrition Series, 2013). The former, which more accurately consist of both *immediate* and *underlying* determinants (UNICEF, 1990), includes age-appropriate feeding, absence of symptoms of diarrhea in the two weeks preceding the survey, mother's receipt of iron supplements, full immunization and deworming medication. Precise definitions are given in Table 1. Diarrhea in the last two weeks obviously cannot have an immediate impact on height measured at the time of the DHS interview. Likewise, the feeding indicators, which are constructed from reported feeding on the day prior to the interview, cannot impact on the individual child's height. Nonetheless, these indicators can be used to compare population level trends in nutrition determinants with trends in nutrition outcomes. Analyses of full immunization and deworming medication are restricted to children aged 12–23 months because these determinants are measurable only from a child's first birthday by when it should have been fully immunized and given deworming medication.

The distal determinants listed in Table 1 are similar to those used by others to explain trends in nutrition outcomes (Headey, 2014; Headey et al., 2015; Headey & Hoddinott, 2014). They include indicators of maternity care, which are underlying determinants (UNICEF, 1990) that have a less direct impact on nutritional status than immunization, deworming medication, feeding practices and iron supplementation. In addition, basic determinants of nutritional status (UNICEF, 1990) are included in this set: birth order, birth interval, mother's height, mother's age, father's education, wealth, drinking water source, sanitation facilities and livestock ownership. Drinking water and sanitation indicators are constructed following the WHO/UNICEF Joint Monitoring Programme categorization of improved and unimproved water sources and sanitation facilities (WHO/UNICEF, 2016). A wealth index is constructed from a principal components analysis of indicators of asset ownership and housing characteristics. Water and sanitation indicators were not included in the principal components analysis because they enter the regression model for nutritional status directly. This analysis is done using observations pooled over the two DHS waves used for each country. The resulting index is used to distinguish the poorest 40 percent of children (over the two waves) from the rest.

We also allow for variation in nutritional status with urban/rural, region, season, child's age and gender.

### 2.2 Methods

For each of the nine countries, we measure the change in each nutritional outcome (mean HAZ and rate of (severe) stunting) between the two DHS surveys conducted in the period from 2005 to 2014. Then, for the seven countries in which there is a significant reduction in stunting, we identify which of the proximal and distal determinants move in directions consistent with having contributed to this decrease. Next, for each of these seven countries, we pool data from the two survey years and regress the outcomes on the distal determinants. The estimated associations are used, along with the changes in the distal determinants, to account for changes in the outcomes. Estimates of means and rates of nutritional outcomes are obtained using all children aged 0–23 months with a valid measure of height. We check that these estimates are robust to using the restricted samples with full item response on all distal determinants that are used for the regression analysis.

Let $Y_{it}$ be a nutritional outcome of child *i* observed in year *t*, and specify this as a linear function of a vector of distal determinants $\;\left(X_{it}\right)$ : $\;Y_{it}=\alpha +X_{it}\beta +\delta YEAR_{t}+\varepsilon_{it}$ , where $YEAR_{t}\;$ is equal to 1 in the later year (*t* = 1) and is 0 in the earlier year (*t* = 0). Then, the change in the mean of the outcome is: ${\bar{Y}}_{1}-{\bar{Y}}_{0}=\left({\bar{X}}_{1}-{\bar{X}}_{0}\right)\hat{\beta }+\hat{\delta }$, where ${\bar{Y}}_{1}$ is the mean outcome in the later year, ${\bar{X}}_{1}$ is the vector of means of the covariates in that year, etc., and $\hat{\beta }$ and $\hat{\delta }$ are least squares estimates. Thus, the change in mean nutritional status is decomposed into a part explained by the changes in the means of distal determinants $\left(\left({\bar{X}}_{1}-{\bar{X}}_{0}\right)\hat{\beta }\right)$ and a part that remains unexplained (δ) (Fortin, 2008). The former can be further decomposed into the contribution of each determinant. The contribution of determinant $X_{k}$ is $\left({\bar{X}}_{k1}-{\bar{X}}_{k0}\right)\beta_{k}$. The standard error of the contribution of each determinant is obtained using the delta method. The unexplained part can arise from changes in the associations between nutritional status and its measured determinants, and from changes in unmeasured determinants.

The same decomposition procedure is used to explain the change in the mean of each proximal determinant. This provides some insight into the pathways through which the distal determinants may possibly affect nutritional status.

It is important to emphasize that this analysis does not identify the causes of changes in nutritional status. The regressions capture associations between the outcome and covariates, which do not necessarily arise from a causal impact of the latter on the former. To avoid obvious endogeneities, we do not regress nutritional outcomes on the proximal determinants. But the distal determinants could also be associated with the outcomes through correlated unobservables. Nonetheless, provided the results are interpreted cautiously, they can be useful in distinguishing determinants that may have contributed to a reduction in stunting from those that are unlikely to have been responsible. The evidence is circumstantial rather than conclusive, if you like.

## 3 Results

### 3.1 Changes in nutritional status

**Fig. 1:**
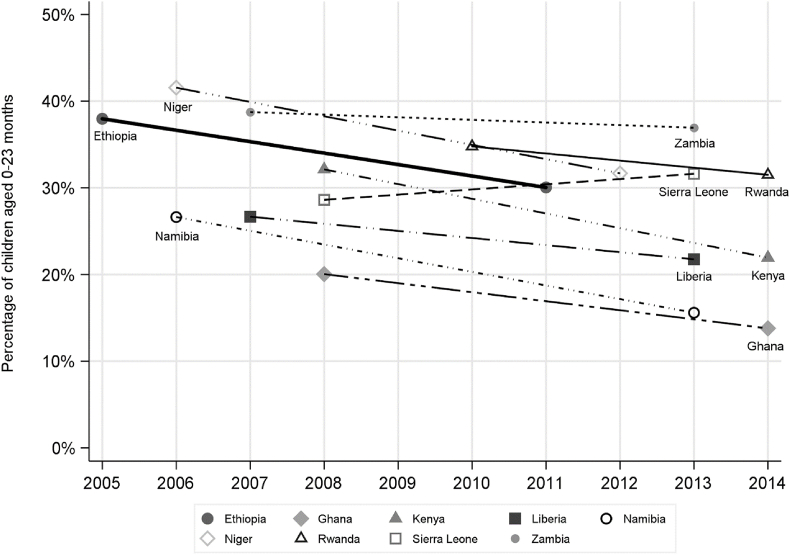
Rates of stunting in 9 Sub-Saharan Africa countries by year, children aged 0–23 months.

The rate of stunting (HAZ < −2) fell by an average of 5.7 percentage points over 6.5 years between 2005 and 2014 in the nine countries (Fig. 1, Table 2). In seven countries (Ethiopia, Ghana, Kenya, Liberia, Namibia, Niger and Rwanda), there was a significant reduction in stunting, which, on average, was equal to 7.3 percentage points over 6.8 years. In these seven countries, there was also a significant reduction in severe stunting (HAZ < −3) and, with the exception of Ghana, the mean HAZ also increased significantly (Table 2).

**Table 2:** Nutritional outcomes by country and year, children aged 0–23 months.

	Mean HAZ		Stunting rate (HAZ < −2)		Severe stunting rate (HAZ < −3)		Sample size	
	First year	Last year	First year	Last year	First year	Last year	First year	Last year
	Mean (SE)	Mean (SE)	Mean (SE)	Mean (SE)	Mean (SE)	Mean (SE)	N	N
*Ethiopia*	−1.25	−1.04***	0.38	0.30***	0.19	0.14***	1531	3813
	(0.05)	(0.03)	(0.01)	(0.01)	(0.01)	(0.01)		
*Ghana*	−0.51	−0.57	0.20	0.14***	0.08	0.04***	1042	1192
	(0.06)	(0.04)	(0.01)	(0.01)	(0.01)	(0.01)		
*Kenya*	−1.11	−0.87***	0.32	0.22***	0.14	0.07***	2164	7652
	(0.04)	(0.02)	(0.01)	(0.00)	(0.01)	(0.02)		
*Liberia*	−0.90	−0.69*	0.27	0.22***	0.11	0.08***	1930	1448
	(0.04)	(0.05)	(0.01)	(0.01)	(0.01)	(0.01)		
*Namibia*	−0.98	−0.47***	0.27	0.16***	0.11	0.06***	1838	885
	(0.04)	(0.06)	(0.01)	(0.01)	(0.01)	(0.01)		
*Niger*	−1.51	−1.16***	0.42	0.32***	0.23	0.15***	1626	1984
	(0.05)	(0.04)	(0.01)	(0.01)	(0.01)	(0.01)		
*Rwanda*	−1.34	−1.23*	0.35	0.32*	0.14	0.12**	1558	1495
	(0.04)	(0.04)	(0.01)	(0.01)	(0.01)	(0.01)		
*Sierra Leone*	−0.84	−0.94	0.29	0.32*	0.17	0.15	971	1771
	(0.07)	(0.05)	(0.01)	(0.01)	(0.01)	(0.01)		
*Zambia*	−1.32	−1.27	0.39	0.37	0.20	0.17***	2267	4734
	(0.04)	(0.03)	(0.01)	(0.01)	(0.01)	(0.01)		

HAZ = height-for-age z-score. Country (first year, last year): Ethiopia (2005, 2011), Ghana (2008, 2014), Kenya (2008, 2014), Liberia (2007, 2013), Namibia (2006, 2013), Niger (2006, 2012), Rwanda (2010, 2014), Sierra Leone (2008, 2013), Zambia (2007, 2013). SE = Robust standard errors. *, ** and *** indicate that the null of no difference between years is rejected at the 10%, 5% and 1%, respectively.

The two countries in which nutritional status did not improve are Zambia and Sierra Leone. The latter is the only country in which the rate of stunting increased significantly (p < 0.1). This is puzzling. The period examined begins six years after the end of the civil war in Sierra Leone, it is before the 2014 Ebola epidemic and it spans years of rapid economic growth. The increase in stunting is observed in both urban and rural areas. However, there is no substantial or significant change in the stunting rate among children aged 0–59 months (37.2%–37.4%). The increase is confined to younger kids, but it is not driven by infants (<6 months) (Appendix Table A1). There is no significant change in the rate of wasting (height-for-weight z-score < −2) among children aged 0–23 months (12%–13%). Mortality selection is a possible explanation for the rise in stunting in Sierra Leone. Over the period examined, the rate of infant mortality fell from 116.9 per 1000 live births in 2008 to 95.8 per 1000 live births in 2013 (World Development Indicators, 2019). This might have reflected greater survival of frail children that were stunted as economic and health conditions improved. As a consequence, the prevalence of stunting increased. If this interpretation is correct, then the rise in stunting would be temporary. It would decline as the health of children improved further and the rate of frail infants dropped.

Restricting the analysis to children aged 6–23 months, and so avoiding the greater measurement error in height among infants (<6 months), has little impact on the trends observed in stunting (Appendix Table A1). As would be expected, stunting rates are higher when the samples are restricted to older children, but the only notable impact on the changes is that the marginally significant (p < 0.1) estimated 3.5 percentage point fall in stunting in Rwanda gains slightly in magnitude but loses significance.

### 3.2 Changes in determinants

**Fig. 2:**
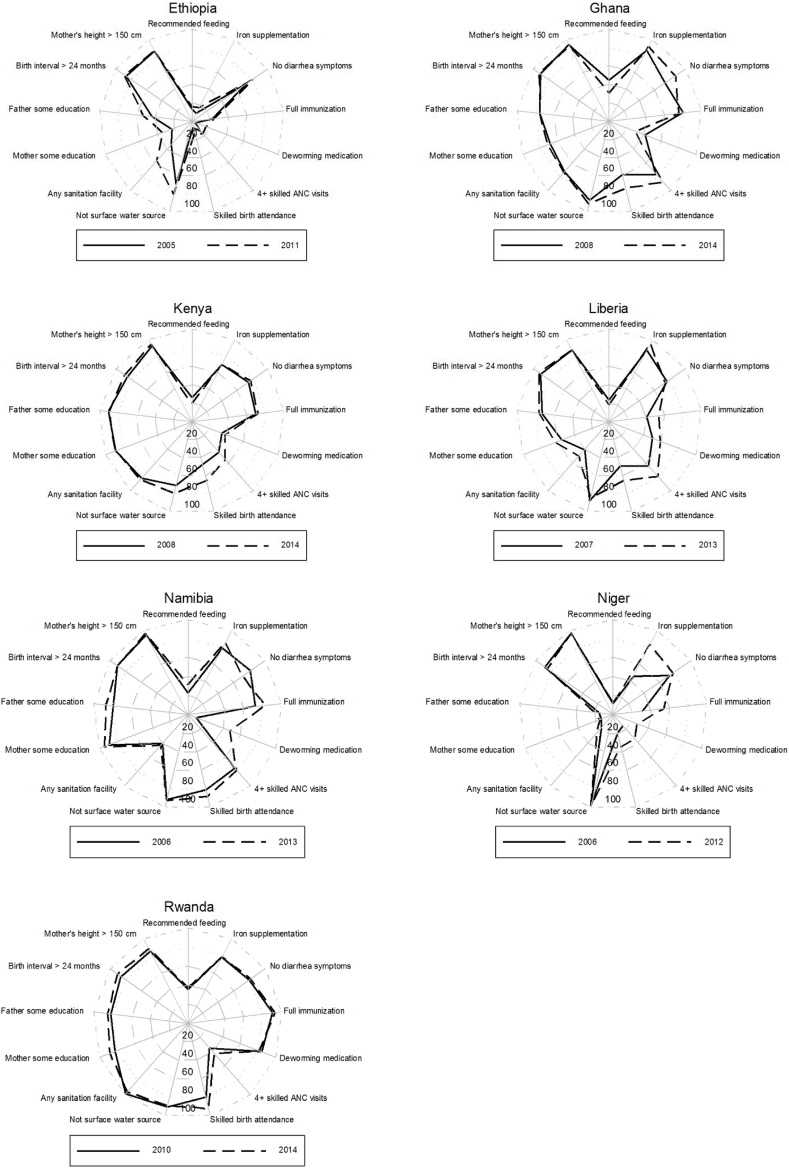
Coverage of determinants of nutritional status by country and year. Deworming medication is not available in the 2006 Niger DHS.

For the seven countries in which there was a significant fall in stunting, the radar charts presented in Fig. 2 facilitate easy comparison of levels of determinants between years and across countries. All determinants are defined such that any impact on nutritional status, if it exists, is positive. Hence, the size of the surface provides an indication of the extent of coverage of nutrition-enhancing factors. Proximal determinants are positioned between 12 o'clock and 4 o'clock. See Appendix Table A3 for the means of all determinants by year and country.

In five of these seven countries (Liberia, Kenya, Namibia, Niger and Rwanda), the proportion of children (aged 12–23 months) who are fully immunized increased significantly (*p < 0.1*). In another group of five countries (Ethiopia, Ghana, Liberia, Namibia and Niger), the proportion of mothers taking iron supplements during pregnancy increased (*p < 0.01*). The proportion of children (aged 12–23 months) receiving deworming medication increased in four countries (*p < 0.05*: Ethiopia, Liberia, Namibia and Kenya). Note that the change cannot be assessed in Niger because deworming medication was not recorded in the 2006 DHS. A significant improvement in all three of these proximal determinants occurred only in Liberia and Namibia. However, these are the only two countries in which the prevalence of diarrhea increased (*p < 0.01*). The rate of this determinant decreased in four countries (*p < 0.01*: Ethiopia, Ghana, Kenya and Niger). Reductions in stunting did not generally coincide with increases in the rate of age-appropriate feeding. Only in Ethiopia (*p < 0.05*) and Namibia (*p < 0.01*) did recommended feeding improve, while deteriorations were observed in Ghana, Kenya and Liberia (*p < 0.01*). The general lack of improvements in appropriate feeding is even more disappointing given that it is the determinant that is typically furthest from full population coverage.

Distal determinants, especially access to maternity care and father's education, improved in most countries. The proportion of mothers who had at least four antenatal care (ANC) visits and delivered under the supervision of a skilled birth attendant increased in all countries (*p < 0.01*) except Namibia, where no change was observed in 4+ ANC visits.

Trends in the other distal determinants -- water sources, sanitation facilities, birth interval and maternal height -- are less clear. The proportion of households relying on sources other than surface water for drinking increased in three countries, while use of any sanitation other than open defecation increased in four countries.

Across countries, coverage of nutrition determinants is most sparse, by some distance, in Ethiopia and Niger.

### 3.3 Association of stunting with distal determinants

Table 3 displays coefficients from linear regressions of the binary indicator of stunting for the seven countries in which this outcome improved significantly. Obviously, these regressions can only be estimated using observations with full item response on all the covariates. Potentially, this introduces a selection bias to the estimated rate of stunting if item non-response is correlated with height. The estimates given in Appendix Table A2 show that this is not the case for six of the seven countries. With the exception of Niger, the levels and changes in stunting are very close to those given in Table 2 that are obtained from the full samples of children aged 0–23 months. In the case of Niger, the 10 percentage point (pp) significant fall in the rate of stunting in the full sample becomes an insignificant 1 pp fall in the restricted sample.^3^ For this country, the decomposition analysis cannot explain the observed fall in stunting. However, there is no such problem for the other six countries.

**Table 3:** Least squares regressions of indicator of stunting of children aged 0–23 months.

	Ethiopia	Ghana	Kenya	Liberia	Namibia	Niger	Rwanda
	Beta (SE)	Beta (SE)	Beta (SE)	Beta (SE)	Beta (SE)	Beta (SE)	Beta (SE)
Mother had 1-3 skilled ANC visits	0.007	−0.058	0.070	−0.023	−0.171	0.013	−0.167
	(0.015)	(0.045)	(0.029)**	(0.034)	(0.058)***	(0.023)	(0.081)**
Mother had 4+ skilled ANC visits	−0.056	−0.040	0.059	−0.003	−0.262	−0.029	−0.190
	(0.019)***	(0.042)	(0.030)**	(0.029)	(0.054)***	(0.026)	(0.082)**
Delivered by skilled birth attendant	−0.079	−0.004	−0.072	−0.014	0.021	0.035	−0.052
	(0.025)***	(0.022)	(0.015)***	(0.021)	(0.039)	(0.022)	(0.026)**
Mother has any education	−0.014	0.043	−0.024	0.040	−0.080	−0.001	−0.019
	(0.016)	(0.023)*	(0.026)	(0.020)**	(0.045)*	(0.026)	(0.025)
Father has any education	−0.020	0.005	0.020	−0.066	0.101	−0.044	−0.041
	(0.014)	(0.026)	(0.028)	(0.022)***	(0.039)**	(0.024)*	(0.024)*
Wealth index in top 60%	−0.039	−0.070	−0.061	−0.043	−0.055	−0.013	−0.067
	(0.013)***	(0.023)***	(0.016)***	(0.024)*	(0.039)	(0.020)	(0.020)***
Surface water not used for drinking	−0.004	0.065	0.003	−0.034	−0.156	−0.111	−0.040
	(0.015)	(0.028)**	(0.016)	(0.027)	(0.053)***	(0.069)	(0.029)
Any sanitation other than open defecation	−0.021	0.026	0.015	−0.039	−0.012	−0.042	−0.014
	(0.014)	(0.025)	(0.020)	(0.020)*	(0.039)	(0.026)	(0.058)
Birth order	−0.001	−0.004	0.012	−0.000	0.009	0.009	0.006
	(0.004)	(0.006)	(0.005)***	(0.006)	(0.010)	(0.006)	(0.007)
Birth interval > 24 months	−0.022	−0.033	0.001	−0.017	0.050	−0.021	−0.025
	(0.019)	(0.030)	(0.018)	(0.030)	(0.043)	(0.024)	(0.026)
Mother taller than 150 cm	−0.102	−0.130	−0.118	−0.104	−0.176	−0.075	−0.149
	(0.018)***	(0.036)***	(0.025)***	(0.027)***	(0.059)***	(0.047)	(0.026)***
Mother's age at birth (in years)	−0.002	0.002	−0.005	−0.004	0.000	−0.006	−0.001
	(0.002)	(0.002)	(0.002)***	(0.002)*	(0.003)	(0.002)***	(0.002)
Have livestock	0.001	0.024	−0.017	0.057	−0.006	NA	−0.028
	(0.022)	(0.019)	(0.015)	(0.019)***	(0.031)	NA	(0.018)
Urban	−0.008	−0.016	−0.029	−0.009	−0.015	−0.122	−0.049
	(0.027)	(0.022)	(0.019)	(0.026)	(0.040)	(0.037)***	(0.032)
Age - 6–11 months	0.115	0.042	0.053	0.045	−0.011	0.059	0.059
	(0.017)***	(0.023)*	(0.017)***	(0.024)*	(0.035)	(0.025)**	(0.024)**
Age - 12–17 months	0.258	0.090	0.189	0.212	0.110	0.230	0.271
	(0.017)***	(0.023)***	(0.017)***	(0.026)***	(0.036)***	(0.025)***	(0.025)***
Age - 18–23 months	0.407	0.227	0.245	0.307	0.219	0.395	0.347
	(0.018)***	(0.024)***	(0.018)***	(0.026)***	(0.037)***	(0.027)***	(0.025)***
Male	0.073	0.040	0.079	0.051	0.078	0.072	0.100
	(0.012)***	(0.016)**	(0.012)***	(0.018)***	(0.025)***	(0.017)***	(0.017)***
Wet season	NA	−0.011	NA	−0.024	NA	0.036	0.065
	NA	(0.017)	NA	(0.037)	NA	(0.020)*	(0.068)
Year = most recent	−0.059	−0.064	−0.074	−0.029	−0.149	−0.139	−0.008
	(0.014)***	(0.017)***	(0.013)***	(0.036)	(0.030)***	(0.022)***	(0.018)
Constant	0.452	0.139	0.371	0.351	0.667	0.580	0.528
	(0.052)***	(0.083)*	(0.056)***	(0.067)***	(0.123)***	(0.123)***	(0.143)***
*R*^2^	0.16	0.09	0.10	0.13	0.15	0.17	0.15
*N*	4993	1845	4795	2084	1015	2875	2603

SE = Robust standard errors; *p < 0.1; **p < 0.05; ***p < 0.01; NA = variable not available. Children aged 0–5 months is reference age category; Mother had 0 skilled ANC visits is reference category. Regressions include region indicators.

The regression coefficients on the year indicator imply that, conditional on the changes occurring in all covariates, the probability that a child is stunted fell by an amount that varied from a 0.8 percentage points in Rwanda (*p < 0.1*) to a very significant 19.6 percentage points in Niger over the period spanned by the respective surveys. Given that the rate of stunting fell by 3.5 percentage points in Rwanda (Table A2), the insignificant year coefficient implies that only 23% ([-0.008/-0.035] × 100) of the reduction in stunting *cannot* be explained by changes in any of the child, maternal and household characteristics controlled for in the regression. So, 77% of the fall can be explained (but is not necessarily caused) by changes in these distal determinants. In the other countries, a much lower fraction of the reduction in stunting can be statistically explained by the changes in covariates -- 3% in Ghana, 13% in Namibia, 23% in Kenya, 29% in Ethiopia and Niger, and 55% in Liberia. For these countries, unmeasured factors explain by far the greatest portion of the fall in stunting.^4^

In all 7 countries, the rate of stunting varies with demographics as anticipated: boys and older children are more likely to be stunted. Except in Namibia and Niger, there is a substantial wealth gap in stunting even after controlling for wealth-related differences in other distal determinants, such as education. Conditional on all else, an Ethiopian child in the top 60% of the wealth distribution is 3.9 percentage points less likely to be stunted than the equivalent child that is among the poorest 40%. The gap is even larger at 7.0, 6.1, 4.3 and 6.7 percentage points in Ghana, Kenya, Liberia and Rwanda, respectively.

Use of maternity care, which changed in a direction consistent with contributing to the reduction in stunting, is sometimes, but not consistently, negatively associated with stunting. The strongest negative association is in Rwanda, where a child delivered by a skilled birth attendant is 5.2 percentage points less likely to be stunted. Conditional even on this, the probability of being stunted is 19 percentage points lower for Rwandan children whose mothers attended four or more ANC sessions compared with those whose mothers had no ANC. These are very large differences relative to the 3.5 percentage point reduction in the rate of stunting in Rwanda. They are consistent with the observed increase in utilization of maternity care having made an important contribution to the reduced rate of stunting. However, in Kenya, use of maternity care is positively associated with stunting. Since it is not plausible that such utilization causes nutritional deficiency, this result is a reminder that the regression coefficients should not be interpreted as estimates of causal effects. Maternity care and stunting are likely to be correlated through unmeasured common factors.

While parental education also generally moved in a direction consistent with having contributed to the reduction in stunting, evidence that it is significantly negatively associated with stunting is rather weak. Except for Ghana and Liberia, maternal education does have a negative regression coefficient, but it is never significant. Paternal education is significantly negatively correlated with stunting only in Liberia, Niger and Rwanda. Counterintuitively, parental education is positively associated with stunting in Namibia. Furthermore, maternal and paternal education are never jointly significant.

Some point estimates are consistent with avoidance of open defecation and drinking surface water both being associated with a lower probability of a child being stunted but none of these relationships reaches statistical significance. Counterintuitively, avoidance of surface water is positively associated with stunting in Ghana.

Taller and older mothers are less likely to have stunted children in most countries. But the fraction of mothers who are taller than 150 cm increased only in Kenya, Namibia and Rwanda and could have contributed to the reduction in stunting only in these four countries. Mother's age increased only in Niger so this is also not a strong candidate to explain the fall in stunting.

In Appendix Tables A5-A11 we show regressions of each proximal determinant of nutritional status on the same distal determinants and covariates used in Table 3. The year effects indicate that, after controlling for all the covariates, unmeasured factors, including vertical programs delivering preventive care, contributed to: reduced prevalence of diarrhea, and increased recommended feeding and iron supplementation (5 countries); and increases deworming (4 countries) and full immunization (3 countries).

Except for recommended feeding in Kenya and Rwanda, mothers receiving iron supplements and children receiving deworming medication in Liberia, there is no significant difference in any of the proximal determinants between children at the top and bottom of the wealth distribution after conditioning on covariates (Appendix Tables A5-A11). It would appear that the wealth gap in stunting that persists even after controlling for differences in covariates does not emerge from wealth-related differences in the observable proximal determinants. Other unobserved determinants explain the differences in the height between the best-off and worst-off children. The most obvious is that the poorer children are less likely to have received adequate food intake and this is partially reflected in the difference in the age-appropriate feeding measure observed in the two countries mentioned at the beginning of this paragraph.

In all countries, ANC and skilled birth attendance are significant positive predictors of full immunization and iron supplementation (Appendix Tables A5-A11). One or other of these two forms of maternity care is associated with a higher likelihood of a child receiving the recommended feeding (4 countries), getting deworming medication (4 countries) and not experiencing diarrhea (3 countries). These patterns are consistent with maternity care impacting on stunting through these proximal determinants.

### 3.4 Explaining the decrease in stunting over time

Having identified characteristics that are associated with stunting, and determined which of these characteristics changed in a direction consistent with a significant fall in stunting, we now put these two pieces of information together to decompose the fall into contributions made by sets of distal determinants and other covariates. The year coefficients in the regressions imply the proportion of the fall in the rate of stunting that *cannot* be explained by changes in covariates. For each country, we will now account for the explained part, which is 3% in Ghana, 13% in Namibia, 23% in Kenya, 29% in Ethiopia and Niger, 55% in Liberia, and 77% in Rwanda.

**Fig. 3:**
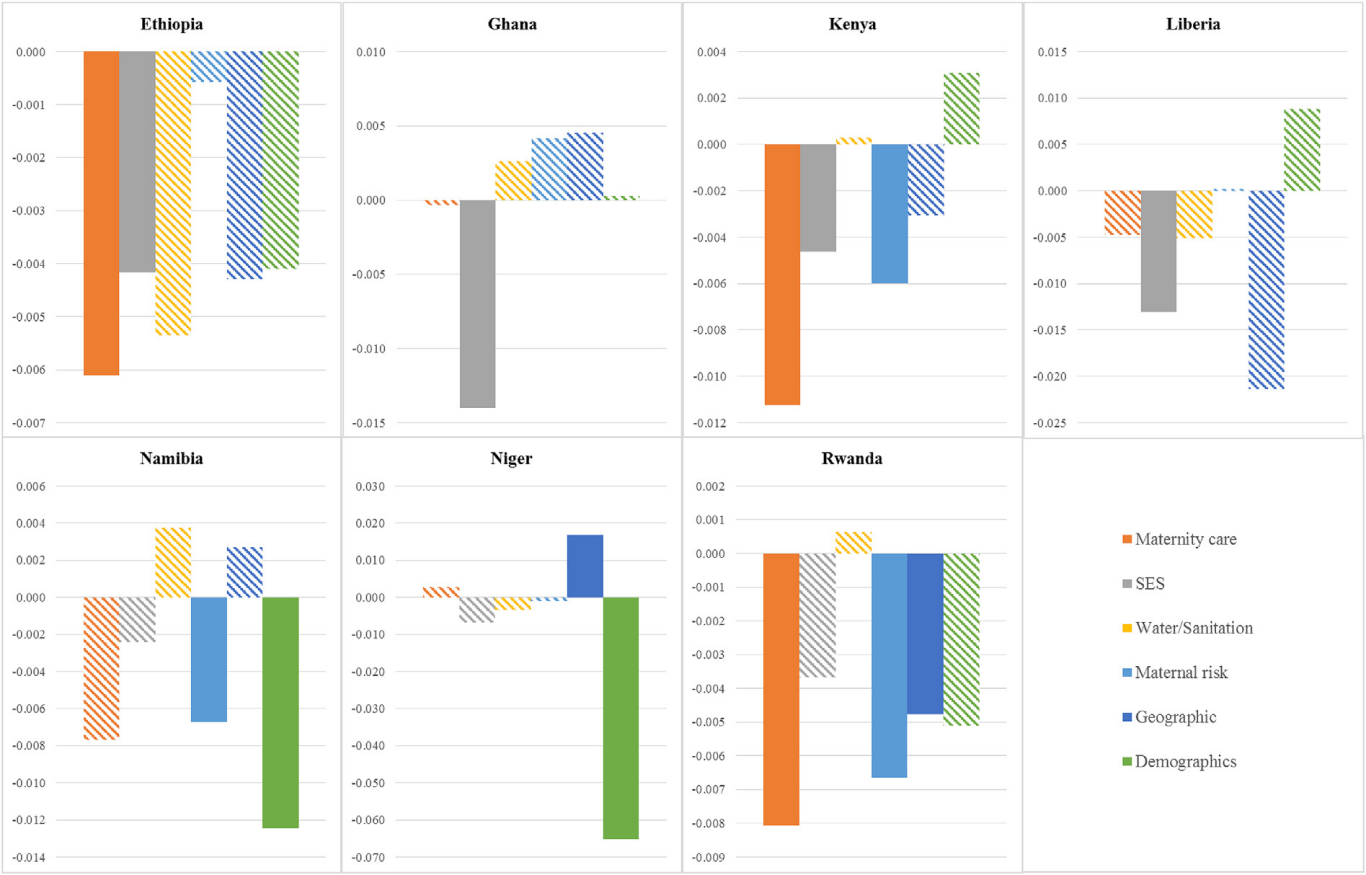
Contributions of changes in distal determinants and covariates to changes in stunting by country, children aged 0–23 months. Significant at 10% level: non-striped shading; Groups of determinants: (1) Maternity care: Mother had 4+ skilled ANC visits, Delivered by skilled birth attendant; (2) Socioeconomic status: Mother has any education, Father has any education, Wealth index in top 60%, Having livestock; (3) Water/Sanitation: Surface water not used for drinking, Any sanitation other than open defecation; (4) Maternal risk: Birth order, Birth interval>24 months, Mother taller than 150 cm, Mother's age at birth; (5) Geographic characteristics: Urban, Regions; (6) Demographics: Child's age, Child is male.

The distal determinants and covariates are grouped into six categories: maternity care, socioeconomic status, water and sanitation, maternal risk characteristics, geographic characteristics, and demographics (see notes to Fig. 3). Fig. 3 shows the contributions of each category to the fall in stunting and whether this fall is statistically significant at the 10% level. Improvements in maternity care account for the largest portion of the explained reduction in the rate of stunting in Ethiopia (24.8%), Kenya (52.3%), and Rwanda (29.2%). This reflects both the positive trends in the use of maternity care and the strong correlation between this care and the likelihood of stunting. Socioeconomic status (SES) and maternal risk characteristics are the next most important contributors to the decrease in stunting. SES is an important contributor in Ethiopia (16.9%), Ghana (523.2%), Kenya (21.4%), and Liberia (37.1%). This is due to substantial reductions of uneducated mothers and fathers combined with the (non-) significant negative correlation between education and stunting. Maternal risk characteristics are important contributors to the reductions in stunting in Kenya (27.8%), Namibia (29.4%), and Rwanda (24.1%). This derives from substantial reductions in the proportion of births with a short birth interval and short maternal height combined with the (non-)significant negative correlation between each of these variables and stunting. The picture in Niger differs from the six other countries: mainly demographics and geographic characteristics have contributed to the fall in stunting between 2006 and 2012. The large contribution of demographics is due to the younger sample of children in 2012 and the significant positive correlation between age and stunting. The substantial relative contribution of geographic factors is the result of a change in the distribution of births across households living in urban or rural areas and across regions.

**Fig. 4:**
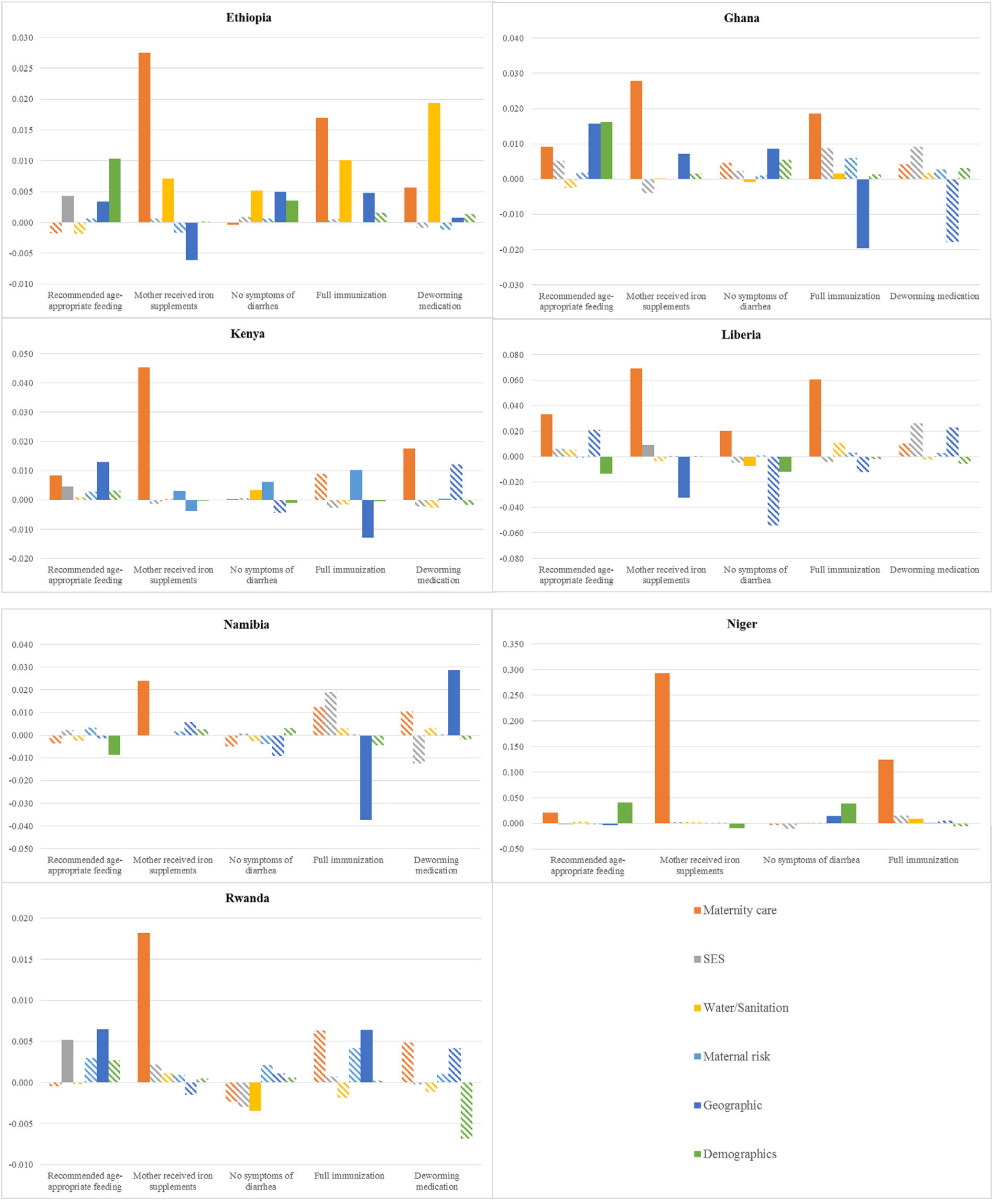
Contributions of changes in distal determinants and covariates to changes in proximal determinants of stunting by country, children aged 0–23 months.

Fig. 4 shows the contributions of distal determinants and other covariates to the change in each of the proximal determinants by country. It should be kept in mind that based on improvements in the mean rates, full immunization, deworming medication, and mother's iron supplementation were identified as the proximal determinants with the highest potential contribution to the fall in stunting, while the contribution of age-appropriate breastfeeding and children free of diarrhea was less clear. Also note that these changes are the same across decompositions of the change in each proximal determinant. What differs are the direction and strength of the association of each proximal determinant with the distal determinants and covariates.

Most striking is the large positive contribution of increased use of maternity care to the rise in the propensity of mothers to take iron supplements in all countries. The contribution of maternity care is due to the strong positive correlations of ANC and skilled birth attendance with the likelihood of iron supplementation in all countries (Appendix Tables A5-A11). For two other proximal determinants -- full immunization and deworming medication -- that displayed strong favorable changes consistent with playing a part in the reduction in stunting, patterns in contributions of sets of distal determinants and covariates are less clear. Maternal risk characteristics are the most important contributors to improvements in full immunization in Kenya and Rwanda, while maternity care is an important contributor to improvements in full immunization in Liberia and Niger.

In Ethiopia and Kenya, the countries in which deworming medication may have been an important contributor to the fall in stunting, at least according to the decomposition analysis, maternity care is an important contributor to the explained change in deworming medication. In addition, in Ethiopia, the largest contribution to the explained change in deworming medication is from water and sanitation (99%). Most of this contribution stems from the strong positive and significant correlation of deworming medication with absence of open defecation, combined with the large reduction in the rate of the latter.

In conclusion, the decomposition analysis reveals that, in a statistical accounting sense, improvements in maternity care make the largest contribution to the reduction in stunting, and to improvements in proximal determinants. This is a result of more mothers having 4+ ANC visits and delivery by a skilled attendant, and the strong negative associations between these indicators and stunting. 5

## 4 Discussion

The proportion of young children (<2 years old) in Sub-Saharan Africa who are stunted fell by 1.3 percentage points annually between 2005 and 2014 on average, in seven out of nine countries with sufficient data available. While this is a welcome improvement in nutritional status that heralds future gains in population health and labour productivity, it is unacceptable that about a third of infants in Ethiopia, Niger and Rwanda, and a fifth of infants in Ghana, Kenya, Liberia and Namibia remain undernourished to an extent that severely impairs their growth. Nonetheless, understanding what has contributed to the achieved reduction in undernutrition in these countries can help us predict whether the trend is likely to continue, speculate on how it may be accelerated and suggest how to possibly replicate it elsewhere.

The fall in stunting in these countries coincides with substantial increases in three proximal determinants: full immunization (5 countries), iron supplements (5 countries) and deworming medication (4 countries). Changes in age-appropriate feeding and prevalence of diarrhea are not consistent with these immediate causes of nutritional status having contributed importantly to the substantial reductions observed in child stunting. Among the distal determinants, use of maternity care and, to a lesser extent, parental education have improved substantially in recent years in most SSA countries examined.

Maternity care emerges from our decomposition analysis as the most important distal determinant associated with reduced stunting, and its effect, if that is indeed what it is, may operate through increased coverage of preventive services like iron supplementation. While it cannot be presumed that this is causal, the result is consistent with what one might expect: increased contact with ANC increases the likelihood that women get and take iron supplements on at least one day during pregnancy. The contribution also derives from the strong positive correlation of iron supplementation with delivery by a skilled professional. The latter cannot possibly increase the likelihood of taking iron supplements during pregnancy. Hence, this correlation, and most likely part of the correlation with the use of ANC, must be due to mothers who have greater opportunities and motivation to use maternity care also being more likely to take iron supplements. It is also possible that access to maternity care increased because more facilities have become available, which highlights the importance of unmeasured factors in explaining the fall in stunting.

Nutritional status can only improve as a result of better dietary intake and reduced experience of disease. Understanding of trends in stunting is constrained by limitations in our ability to measure these biological determinants within the context of large surveys. The DHS uses a recall period of only 24 h to record food intake and gathers information only on the types of food consumed and not their quantities. These limitations make it impossible to relate nutrition outcomes to energy inputs at the child level. Reliance on reported illness may also induce bias if the respondent omits to mention episodes of sickness that did not result in the utilization of medical care or medicines.

The DHS does not record the duration of breastfeeding. We approximated this by calculating the median age of the children (0–23 months) currently breastfed. In every survey year, this was 9–11 months, suggesting that there has been no substantial change in the length of time that infants are breastfed and, consequently, that this is unlikely to have contributed to the reduction in the rate of stunting.

The DHS does not seek to measure household living standards by collecting detailed data on consumption. It relies on asset ownership and housing conditions to construct a proxy index of wealth. This is useful in distinguishing between socioeconomic groups and measuring inequality in nutritional status between them. However, it is not very sensitive to changes in living standards over time. As a result of relying on this measure, we have underestimated the contribution of improved economic conditions to gains in the nutritional status of children. Its effect will be part of the unexplained reduction in stunting in our decompositions.

## 5 Conclusions

Our analysis suggests that the fall in stunting in Sub-Saharan Africa has not been driven by better feeding practices, nor by reduced prevalence of diarrhea. Improved access to maternity care does correlate with improved nutritional status. Given the potential for correlated unobservable determinants of both nutritional status and maternity care, our decomposition analysis does not identify the contribution of the latter to improvements in the former. But the circumstantial evidence it offers is at least strong enough to warrant more detailed investigation of the question of whether maternity care is potentially a channel through which to target further attacks on the blight of undernourished children.

## Funding

This work was supported by UNICEF [RFPS-USA-2015-502132: Analysis of nationally representative surveys to examine the determinants of stunting in selected low- and middle-income countries].

## Ethical statement

Ethics approval is not required because the data is not collected from human subjects and we use secondary data.

## Declarations of interest

None.

## Appendix A Supplementary data

Supplementary material - Appendix_V2 Supplementary material - Appendix_V2

## Footnotes

1 For this reason, Malawi and Mozambique – two of the countries that have not experienced improvements in child nutritional status – are not included.

2 There is an ongoing debate about the extent to which stunting by the age of 2 years is irreversible (see Behrman (2016) for a review). Studies by Addo et al., 2015, Martorell et al., 2010, and Thurow, 2016 found t stunting to become irreversible by the age of 2 years. This older belief in irreversibility has been challenged by some more recent studies. For example, Lundeen et al. (2014) show that there is more catch-up growth in height than many had previously assumed.

3 This is because stunting is substantially higher among children with missing information on the covariates in the earlier year.

4 The regressions presented in Table A4 obtained from the samples of children aged 6–23 months reveal that the unexplained part of the changes in stunting (shown by the year coefficients) is somewhat larger compared with the estimates obtained from children aged 0–23 months (Table 3).

5 Appendix Fig. A1 presents estimates of the decomposition of changes in mean height-for-age z-scores. The decomposition results for rates of stunting are generally robust to dropping children aged 0–5 months (Appendix Fig. A2). One notable difference is that the contributions of demographics in Ethiopia and Ghana are larger than are obtained from the sample of children aged 0–23 months (Fig. 3).
