# Supplementary material for: What explains the fall in child stunting in Sub-Saharan Africa?
Source: SSM Popul Health. 2019 May 13;8:100384. doi: 10.1016/j.ssmph.2019.100384 (PMC6545382; doi:10.1016/j.ssmph.2019.100384)
Supplement: Supplementary material - Appendix_V2 [file mmc1.docx]

# Supplementary material - Appendix

Table A1. Nutritional outcomes by country and year, children aged 6-23 months, robustness check

|  | **Mean HAZ** | | **Stunting rate (HAZ<-2)** | | **Severe stunting rate (HAZ<-3)** | | **Sample size** | |
| --- | --- | --- | --- | --- | --- | --- | --- | --- |
|  | *First year* | *Last year* | *First year* | *Last year* | *First year* | *Last year* | *First year* | *Last year* |
|  | *Mean (SE)* | *Mean (SE)* | *Mean (SE)* | *Mean (SE)* | *Mean (SE)* | *Mean (SE)* | *N* | *N* |
| *Ethiopia* | -1.61 | -1.38*** | 0.45 | 0.36*** | 0.23 | 0.17*** | 1,177 | 2,770 |
|  | (0.06) | (0.03) | (0.01) | (0.01) | (0.01) | (0.01) |  |  |
| *Ghana* | -0.75 | -0.75 | 0.23 | 0.16*** | 0.08 | 0.04*** | 809 | 875 |
|  | (0.06) | (0.05) | (0.01) | (0.01) | (0.01) | (0.01) |  |  |
| *Kenya* | -1.35 | -1.03*** | 0.37 | 0.25*** | 0.15 | 0.08*** | 1,643 | 5,900 |
|  | (0.04) | (0.02) | (0.01) | (0.01) | (0.01) | (0.00) |  |  |
| *Liberia* | -1.15 | -0.86*** | 0.31 | 0.25*** | 0.13 | 0.09*** | 1,466 | 1,114 |
|  | (0.05) | (0.05) | (0.01) | (0.01) | (0.01) | (0.01) |  |  |
| *Namibia* | -1.16 | -0.62*** | 0.30 | 0.17*** | 0.12 | 0.06** | 1,362 | 640 |
|  | (0.04) | (0.07) | (0.01) | (0.01) | (0.01) | (0.01) |  |  |
| *Niger* | -1.97 | -1.47*** | 0.51 | 0.37*** | 0.28 | 0.17*** | 1,177 | 1,427 |
|  | (0.05) | (0.05) | (0.01) | (0.01) | (0.01) | (0.01) |  |  |
| *Rwanda* | -1.57 | -1.44** | 0.40 | 0.36 | 0.17 | 0.13** | 1,200 | 1,154 |
|  | (0.04) | (0.04) | (0.01) | (0.01) | (0.01) | (0.01) |  |  |
| *Sierra Leone* | -1.06 | -1.03 | 0.32 | 0.34* | 0.19 | 0.16 | 742 | 1,361 |
|  | (0.08) | (0.06) | (0.02) | (0.01) | (0.01) | (0.01) |  |  |
| *Zambia* | -1.56 | -1.54 | 0.44 | 0.43 | 0.22 | 0.20*** | 1,753 | 3,651 |
|  | (0.05) | (0.03) | (0.01) | (0.01) | (0.01) | (0.01) |  |  |

HAZ = height-for-age z-score. Country (first year, last year): Ethiopia (2005, 2011), Ghana (2008, 2014), Kenya (2008, 2014), Liberia (2007, 2013), Namibia (2006, 2013), Niger (2006, 2012), Rwanda (2010, 2014), Sierra Leone (2008, 2013), Zambia (2007, 2013). SE = Robust standard errors. *, ** and *** indicate that the null of no difference between years is rejected at the 10%, 5% and 1%, respectively.

Table A2. Nutritional outcomes by country and year based on restricted sample used in regressions, children aged 0-23 months

|  | **Mean HAZ** | | **Stunting rate (HAZ<-2)** | | **Severe stunting rate (HAZ<-3)** | | **Sample size** | |
| --- | --- | --- | --- | --- | --- | --- | --- | --- |
|  | *First year* | *Last year* | *First year* | *Last year* | *First year* | *Last year* | *First year* | *Last year* |
|  | *Mean (SE)* | *Mean (SE)* | *Mean (SE)* | *Mean (SE)* | *Mean (SE)* | *Mean (SE)* | *N* | *N* |
| *Ethiopia* | -1.25 | -1.03*** | 0.38 | 0.30*** | 0.19 | 0.14*** | 1,426 | 3,567 |
|  | (0.05) | (0.03) | (0.01) | (0.01) | (0.01) | (0.01) |  |  |
| *Ghana* | -0.47 | -0.51 | 0.19 | 0.13*** | 0.07 | 0.03*** | 838 | 1,007 |
|  | (0.07) | (0.04) | (0.01) | (0.01) | (0.01) | (0.01) |  |  |
| *Kenya* | -1.09 | -0.91*** | 0.32 | 0.22*** | 0.13 | 0.08*** | 1,710 | 3,085 |
|  | (0.05) | (0.03) | (0.01) | (0.01) | (0.01) | (0.00) |  |  |
| *Liberia* | -0.87 | -0.63 | 0.26 | 0.20*** | 0.11 | 0.07*** | 1,146 | 938 |
|  | (0.06) | (0.06) | (0.01) | (0.01) | (0.01) | (0.01) |  |  |
| *Namibia* | -0.99 | -0.39*** | 0.28 | 0.11*** | 0.12 | 0.04*** | 757 | 258 |
|  | (0.06) | (0.10) | (0.02) | (0.02) | (0.01) | (0.01) |  |  |
| *Niger* | -1.96 | -1.15*** | 0.51 | 0.31*** | 0.28 | 0.14*** | 1,097 | 1,778 |
|  | (0.06) | (0.04) | (0.02) | (0.01) | (0.01) | (0.01) |  |  |
| *Rwanda* | -1.34 | -1.21* | 0.35 | 0.31* | 0.14 | 0.11** | 1,326 | 1,277 |
|  | (0.04) | (0.04) | (0.01) | (0.01) | (0.01) | (0.01) |  |  |

HAZ = height-for-age z-score. Country (first year, last year): Ethiopia (2005, 2011), Ghana (2008, 2014), Kenya (2008, 2014), Liberia (2007, 2013), Namibia (2006, 2013), Niger (2006, 2012), Rwanda (2010, 2014), Sierra Leone (2008, 2013), Zambia (2007, 2013). SE = Robust standard errors. *, ** and *** indicate that the null of no difference between years is rejected at the 10%, 5% and 1%, respectively.

Table A3. Means of variables for children aged 0-23 months and 12-23 months

| Variable | Ethiopia | | Ghana | | Kenya | | Liberia | | Namibia | | Niger | | Rwanda | |
| --- | --- | --- | --- | --- | --- | --- | --- | --- | --- | --- | --- | --- | --- | --- |
| Year | 2005 | 2011 | 2008 | 2014 | 2008 | 2014 | 2007 | 2013 | 2006 | 2013 | 2006 | 2012 | 2010 | 2014 |
|  | *Mean (N)* | *Mean (N)* | *Mean (N)* | *Mean (N)* | *Mean (N)* | *Mean (N)* | *Mean (N)* | *Mean (N)* | *Mean (N)* | *Mean (N)* | *Mean (N)* | *Mean (N)* | *Mean (N)* | *Mean (N)* |
| Poor age-appropriate feeding | 0.46 (1,529) | 0.43 (3,809) | 0.18 (1,040) | 0.26 (1,192) | 0.32 (2,164) | 0.58 (6,730) | 0.29 (1,928) | 0.26 (1,446) | 0.42 (1,833) | 0.31 (885) | 0.51 (1,212) | 0.41 (1,984) | 0.18 (1,557) | 0.19 (1,494) |
| Partial age-appropriate feeding | 0.40 (1,529) | 0.41 (3,809) | 0.38 (1,040) | 0.43 (1,192) | 0.43 (2,164) | 0.22 (6,730) | 0.48 (1,928) | 0.56 (1,446) | 0.35 (1,833) | 0.37 (885) | 0.38 (1,212) | 0.46 (1,984) | 0.47 (1,557) | 0.44 (1,494) |
| Recommended age-appropriate feeding | 0.13 (1,529) | 0.16 (3,809) | 0.45 (1,040) | 0.31 (1,192) | 0.26 (2,164) | 0.20 (6,730) | 0.23 (1,928) | 0.18 (1,446) | 0.23 (1,833) | 0.32 (885) | 0.12 (1,212) | 0.13 (1,984) | 0.35 (1,557) | 0.37 (1,494) |
| Mother received iron supplements | 0.10 (1,477) | 0.17 (3,681) | 0.87 (952) | 0.92 (1,113) | 0.69 (1,907) | 0.70 (3,465) | 0.87 (1,491) | 0.96 (1,210) | 0.79 (1,491) | 0.86 (647) | 0.45 (1,550) | 0.84 (1,863) | 0.78 (1,490) | 0.79 (1,446) |
| No symptoms of diarrhea | 0.73 (1,529) | 0.80 (3,808) | 0.73 (1,041) | 0.87 (1,192) | 0.74 (2,164) | 0.77 (7,649) | 0.76 (1,922) | 0.73 (1,445) | 0.81 (1,834) | 0.71 (883) | 0.73 (1,625) | 0.77 (1,984) | 0.80 (1,558) | 0.81 (1,495) |
| Full immunization^#^ | 0.23 (766) | 0.24 (1,786) | 0.80 (520) | 0.76 (582) | 0.69 (1,035) | 0.72 (3,871) | 0.41 (887) | 0.54 (659) | 0.72 (851) | 0.83 (407) | 0.31 (751) | 0.54 (890) | 0.90 (783) | 0.93 (732) |
| Deworming medication^#^ | 0.04 (742) | 0.16 (1,780) | 0.42 (519) | 0.30 (581) | 0.35 (1,035) | 0.39 (1,902) | 0.50 (884) | 0.59 (660) | 0.10 (843) | 0.48 (393) | NA | 0.27 (884) | 0.84 (783) | 0.82 (732) |
| Mother had 1-3 skilled ANC visits | 0.18 (1,493) | 0.26 (3,716) | 0.18 (999) | 0.10 (1,143) | 0.47 (2,036) | 0.41 (7,395) | 0.13 (1,683) | 0.17 (1,345) | 0.19 (1,664) | 0.18 (674) | 0.33 (1,588) | 0.53 (1,920) | 0.63 (1,517) | 0.57 (1,460) |
| Mother had 4+ skilled ANC visits | 0.13 (1,493) | 0.18 (3,716) | 0.76 (999) | 0.87 (1,143) | 0.44 (2,036) | 0.55 (7,395) | 0.64 (1,683) | 0.79 (1,345) | 0.75 (1,664) | 0.78 (674) | 0.18 (1,588) | 0.34 (1,920) | 0.35 (1,517) | 0.42 (1,460) |
| Delivered by skilled birth attendant | 0.07 (1,531) | 0.12 (3,813) | 0.59 (999) | 0.74 (1,192) | 0.48 (2,164) | 0.66 (7,395) | 0.49 (1,683) | 0.66 (1,448) | 0.81 (1,836) | 0.89 (885) | 0.20 (1,626) | 0.34 (1,984) | 0.80 (1,558) | 0.92 (1,494) |
| Mother has any education | 0.23 (1,531) | 0.33 (3,813) | 0.68 (1,042) | 0.71 (1,192) | 0.88 (2,164) | 0.89 (7,652) | 0.55 (1,928) | 0.62 (1,448) | 0.89 (1,838) | 0.94 (885) | 0.14 (1,626) | 0.15 (1,982) | 0.82 (1,558) | 0.88 (1,495) |
| Father has any education | 0.44 (1,510) | 0.53 (3,750) | 0.76 (941) | 0.76 (1,088) | 0.91 (1,982) | 0.90 (3,389) | 0.73 (1,566) | 0.77 (1,159) | 0.79 (1,007) | 0.87 (399) | 0.16 (1,573) | 0.20 (1,932) | 0.82 (1,408) | 0.85 (1,328) |
| Wealth index in top 60%* | 0.52 (1,531) | 0.55 (3,813) | 0.41 (1,042) | 0.60 (1,192) | 0.42 (2,164) | 0.48 (7,652) | 0.46 (1,930) | 0.65 (1,448) | 0.44 (1,838) | 0.53  (885) | 0.39 (1,626) | 0.77 (1,984) | 0.55 (1,558) | 0.60 (1,494) |
| Surface water source (unimproved) | 0.31 (1,527) | 0.19 (3,799) | 0.13 (1,041) | 0.08 (1,192) | 0.29 (2,156) | 0.20 (7,649) | 0.12 (1,903) | 0.16 (1,448) | 0.08 (1,810) | 0.07 (868) | 0.01 (1,619) | 0.01 (1,984) | 0.10 (1,537) | 0.11 (1,494) |
| Other unimproved water source (other than surface water) | 0.12 (1,527) | 0.35 (3,799) | 0.10 (1,041) | 0.24 (1,192) | 0.14 (2,156) | 0.15 (7,649) | 0.22 (1,903) | 0.14 (1,448) | 0.06 (1,810) | 0.09 (868) | 0.60 (1,619) | 0.33 (1,984) | 0.18 (1,537) | 0.15 (1,494) |
| Improved water source | 0.57 (1,527) | 0.46 (3,799) | 0.77 (1,041) | 0.67 (1,192) | 0.57 (2,156) | 0.65 (7,649) | 0.66 (1,903) | 0.71 (1,448) | 0.86 (1,810) | 0.84 (868) | 0.39 (1,619) | 0.66 (1,984) | 0.72 (1,537) | 0.74 (1,494) |
| Open defecation (unimproved) | 0.66 (1,529) | 0.43 (3,812) | 0.27 (1,041) | 0.26 (1,192) | 0.18 (2,164) | 0.17 (7,649) | 0.60 (1,924) | 0.50 (1,446) | 0.60 (1,838) | 0.57 (883) | 0.82 (1,626) | 0.73 (1,983) | 0.01 (1,558) | 0.04 (1,495) |
| Other unimproved sanitation facility (other than open defecation) | 0.29 (1,529) | 0.50 (3,812) | 0.67 (1,041) | 0.63 (1,192) | 0.63 (2,164) | 0.63 (7,649) | 0.34 (1,924) | 0.38 (1,446) | 0.14 (1,838) | 0.17 (883) | 0.07 (1,626) | 0.17 (1,983) | 0.41 (1,558) | 0.44 (1,495) |
| Improved sanitation facility | 0.04 (1,529) | 0.07 (3,812) | 0.06 (1,041) | 0.11 (1,192) | 0.18 (2,164) | 0.20 (7,649) | 0.07 (1,924) | 0.12 (1,446) | 0.26 (1,838) | 0.26 (883) | 0.11 (1,626) | 0.09 (1,983) | 0.57 (1,558) | 0.52 (1,495) |
| Birth order | 4.24 (1,531) | 4.04 (3,813) | 3.21 (1,042) | 3.28 (1,192) | 3.46 (2,164) | 3.15 (7,652) | 3.56 (1,930) | 3.34 (1,448) | 2.79 (1,838) | 2.74 (885) | 4.52 (1,626) | 4.65 (1,984) | 3.25 (1,558) | 3.04 (1,495) |
| Birth interval > 24 months | 0.86 (1,531) | 0.88 (3,813) | 0.90 (1,042) | 0.92 (1,192) | 0.85 (2,164) | 0.88 (7,652) | 0.90 (1,930) | 0.92 (1,448) | 0.91 (1,838) | 0.90 (885) | 0.88 (1,626) | 0.84 (1,984) | 0.86 (1,558) | 0.90 (1,495) |
| Mother taller than 150 cm | 0.86 (1,526) | 0.86 (3,799) | 0.94 (1,036) | 0.93 (1,191) | 0.93 (2,152) | 0.94 (3,700) | 0.87 (1,920) | 0.88 (1,443) | 0.95 (1,824) | 0.96 (869) | 0.96 (1,621) | 0.97 (1,965) | 0.86 (1,557) | 0.89 (1,492) |
| Mother’s age at birth (in years) | 27.39 (1,531) | 27.07 (3,813) | 27.58 (1,042) | 28.28 (1,192) | 26.00 (2,164) | 26.13 (7,652) | 26.63 (1,930) | 25.91 (1,448) | 26.73 (1,838) | 26.96 (885) | 26.69 (1,626) | 27.08 (1,984) | 27.90 (1,558) | 28.23 (1,495) |
| Having livestock | 0.88 (1,531) | 0.86 (3,813) | 0.49 (1,042) | 0.46 (1,192) | 0.73 (2,164) | 0.70 (7,652) | 0.40 (1,930) | 0.41 (1,448) | 0.52 (1,835) | 0.55 (885) | NA | 0.74 (1,984) | 0.56 (1,558) | 0.48 (1,495) |
| Urban | 0.08 (1,531) | 0.13 (3,813) | 0.38 (1,042) | 0.45 (1,192) | 0.19 (2,164) | 0.34 (7,652) | 0.31 (1,930) | 0.51 (1,448) | 0.38 (1,838) | 0.41 (885) | 0.14 (1,626) | 0.14 (1,984) | 0.12 (1,558) | 0.18 (1,495) |
| Wet season | 0.41 (1,531) | 1.00 (3,813) | 0.41 (1,042) | 0.99 (1,192) | 0.00 (2,164) | 0.95 (7,652) | 0.08 (1,930) | 0.89 (1,448) | 1.00 (1,838) | 0.00 (885) | 0.54 (1,626) | 0.76 (1,984) | 0.98 (1,558) | 0.99 (1,495) |
| Child’s age (0-5 months) | 0.25 (1,531) | 0.27 (3,813) | 0.22 (1,042) | 0.26 (1,192) | 0.23 (2,164) | 0.23 (7,652) | 0.24 (1,930) | 0.21 (1,448) | 0.26 (1,838) | 0.27 (885) | 0.28 (1,626) | 0.31 (1,984) | 0.23 (1,558) | 0.23 (1,495) |
| Child’s age (6-11 months) | 0.27 (1,531) | 0.27 (3,813) | 0.27 (1,042) | 0.25 (1,192) | 0.27 (2,164) | 0.26 (7,652) | 0.29 (1,930) | 0.31 (1,448) | 0.27 (1,838) | 0.27 (885) | 0.26 (1,626) | 0.25 (1,984) | 0.27 (1,558) | 0.28 (1,495) |
| Child’s age (12-17 months) | 0.29 (1,531) | 0.24 (3,813) | 0.28 (1,042) | 0.25 (1,192) | 0.24 (2,164) | 0.27 (7,652) | 0.22 (1,930) | 0.26 (1,448) | 0.26 (1,838) | 0.24 (885) | 0.26 (1,626) | 0.26 (1,984) | 0.24 (1,558) | 0.26 (1,495) |
| Child’s age (18-23 months) | 0.19 (1,531) | 0.22 (3,813) | 0.22 (1,042) | 0.24 (1,192) | 0.25 (2,164) | 0.24 (7,652) | 0.24 (1,930) | 0.22 (1,448) | 0.21 (1,838) | 0.21 (885) | 0.20 (1,626) | 0.19 (1,984) | 0.26 (1,558) | 0.23 (1,495) |
| Child is male | 0.51 (1,531) | 0.51 (3,813) | 0.49 (1,042) | 0.50 (1,192) | 0.52 (2,164) | 0.50 (7,652) | 0.53 (1,930) | 0.56 (1,448) | 0.52 (1,838) | 0.50 (885) | 0.50 (1,626) | 0.50 (1,984) | 0.49 (1,558) | 0.49 (1,495) |

Unless otherwise noted, all children are under the age of 2, all mothers are aged 15 to 49 and had a birth in the past 2 years. ^#^Children aged 12-23 months. Data on feeding, antenatal care and iron supplements only collected for the youngest child. Sample weights were used to produce all results. NA = not available. *Based on pooled wealth index, which is produced by pooling data from the two most recent surveys for each country on household’s assets ownership (note: pooled wealth index was constructed without water and sanitation components).

Table A4. Least squares regressions of indicator of stunting of children aged 6-23 months, robustness check

|  | **Ethiopia** | **Ghana** | **Kenya** | **Liberia** | **Namibia** | **Niger** | **Rwanda** |
| --- | --- | --- | --- | --- | --- | --- | --- |
|  | *Beta (SE)* | *Beta (SE)* | *Beta (SE)* | *Beta (SE)* | *Beta (SE)* | *Beta (SE)* | *Beta (SE)* |
| Mother had 1-3 skilled ANC visits | -0.011 | -0.130 | 0.069 | -0.085 | -0.117 | 0.022 | -0.223 |
|  | (0.020) | (0.057)** | (0.035)** | (0.041)** | (0.070)* | (0.026) | (0.093)** |
| Mother had 4+ skilled ANC visits | -0.065 | -0.089 | 0.059 | -0.047 | -0.218 | -0.014 | -0.260 |
|  | (0.024)*** | (0.053)* | (0.035)* | (0.035) | (0.066)*** | (0.029) | (0.094)*** |
| Delivered by skilled birth attendant | -0.077 | 0.006 | -0.084 | -0.022 | 0.030 | 0.050 | -0.053 |
|  | (0.031)** | (0.027) | (0.018)*** | (0.025) | (0.048) | (0.026)* | (0.031)* |
| Mother has any education | -0.022 | 0.095 | -0.031 | 0.015 | -0.120 | -0.012 | -0.018 |
|  | (0.019) | (0.029)*** | (0.030) | (0.024) | (0.055)** | (0.029) | (0.030) |
| Father has any education | -0.029 | -0.023 | 0.018 | -0.063 | 0.164 | -0.068 | -0.060 |
|  | (0.018) | (0.032) | (0.033) | (0.027)** | (0.047)*** | (0.027)** | (0.028)** |
| Wealth index in top 60% | -0.052 | -0.088 | -0.062 | -0.043 | -0.082 | -0.005 | -0.083 |
|  | (0.017)*** | (0.028)*** | (0.020)*** | (0.029) | (0.047)* | (0.023) | (0.023)*** |
| Surface water not used for drinking | 0.008 | 0.080 | 0.009 | -0.047 | -0.171 | -0.151 | -0.044 |
|  | (0.019) | (0.035)** | (0.019) | (0.033) | (0.063)*** | (0.075)** | (0.034) |
| Any sanitation other than open defecation | -0.026 | 0.024 | 0.027 | -0.043 | -0.060 | -0.055 | -0.088 |
|  | (0.017) | (0.032) | (0.024) | (0.025)* | (0.047) | (0.030)* | (0.070) |
| Birth order | 0.001 | 0.001 | 0.015 | -0.000 | 0.007 | 0.009 | 0.009 |
|  | (0.005) | (0.008) | (0.005)*** | (0.008) | (0.012) | (0.006) | (0.008) |
| Birth interval > 24 months | -0.008 | -0.054 | 0.009 | -0.031 | 0.098 | -0.019 | -0.024 |
|  | (0.023) | (0.037) | (0.022) | (0.037) | (0.051)* | (0.027) | (0.030) |
| Mother taller than 150 cm | -0.097 | -0.122 | -0.138 | -0.106 | -0.187 | -0.057 | -0.178 |
|  | (0.023)*** | (0.045)*** | (0.030)*** | (0.032)*** | (0.071)*** | (0.053) | (0.031)*** |
| Mother’s age at birth (in years) | -0.002 | 0.001 | -0.007 | -0.005 | 0.001 | -0.007 | -0.001 |
|  | (0.002) | (0.002) | (0.002)*** | (0.003)** | (0.003) | (0.003)** | (0.003) |
| Have livestock | -0.005 | 0.021 | -0.022 | 0.062 | -0.004 | NA | -0.024 |
|  | (0.028) | (0.023) | (0.018) | (0.023)*** | (0.038) | NA | (0.021) |
| Urban | -0.013 | -0.010 | -0.039 | -0.008 | 0.014 | -0.142 | -0.060 |
|  | (0.033) | (0.027) | (0.022)* | (0.032) | (0.048) | (0.042)*** | (0.037) |
| Age - 12-17 months | 0.142 | 0.047 | 0.137 | 0.167 | 0.119 | 0.169 | 0.214 |
|  | (0.018)*** | (0.024)* | (0.017)*** | (0.026)*** | (0.036)*** | (0.022)*** | (0.025)*** |
| Age - 18-23 months | 0.292 | 0.186 | 0.193 | 0.257 | 0.234 | 0.337 | 0.287 |
|  | (0.019)*** | (0.025)*** | (0.018)*** | (0.026)*** | (0.037)*** | (0.024)*** | (0.025)*** |
| Male | 0.097 | 0.032 | 0.090 | 0.082 | 0.076 | 0.074 | 0.102 |
|  | (0.015)*** | (0.020) | (0.014)*** | (0.022)*** | (0.030)** | (0.019)*** | (0.020)*** |
| Wet season | NA | -0.001 | NA | -0.031 | NA | 0.052 | 0.045 |
|  | NA | (0.022) | NA | (0.045) | NA | (0.022)** | (0.076) |
| Year=most recent | -0.074 | -0.074 | -0.095 | -0.035 | -0.168 | -0.154 | -0.005 |
|  | (0.017)*** | (0.021)*** | (0.016)*** | (0.043) | (0.036)*** | (0.023)*** | (0.021) |
| Constant | 0.585 | 0.221 | 0.478 | 0.478 | 0.520 | 0.669 | 0.754 |
|  | (0.064)*** | (0.102)** | (0.067)*** | (0.081)*** | (0.150)*** | (0.134)*** | (0.168)*** |
| *R*^2^ | 0.13 | 0.09 | 0.09 | 0.13 | 0.18 | 0.16 | 0.13 |
| *N* | 3,674 | 1,388 | 3,705 | 1,566 | 759 | 2,325 | 2,003 |

SE = Robust standard errors; * p<0.1; ** p<0.05; *** p<0.01; NA = variable not available. Children aged 6-11 months is reference age category; Mother had 0 skilled ANC visits is reference category. Regressions include region indicators.

Table A5. Least squares regressions of indicators of HAZ and proximal determinants of nutritional status of children aged 0-23 and 12-23 months from Ethiopia

|  | **Children aged 0-23 months** | | | | **Children aged 12-23 months** | |
| --- | --- | --- | --- | --- | --- | --- |
|  | **HAZ** | **Recommended age-appropriate feeding** | **Mother received iron supplements** | **No symptoms of diarrhea** | **Full immunization** | **Deworming medication** |
|  | *Beta (SE)* | *Beta (SE)* | *Beta (SE)* | *Beta (SE)* | *Beta (SE)* | *Beta (SE)* |
| Mother had 1-3 skilled ANC visits | -0.099 | -0.007 | 0.170 | -0.049 | 0.151 | 0.053 |
|  | (0.063) | (0.011) | (0.012)*** | (0.015)*** | (0.021)*** | (0.017)*** |
| Mother had 4+ skilled ANC visits | 0.292 | -0.002 | 0.272 | 0.031 | 0.174 | 0.122 |
|  | (0.078)*** | (0.014) | (0.015)*** | (0.018)* | (0.025)*** | (0.020)*** |
| Delivered by skilled birth attendant | 0.314 | -0.021 | 0.036 | 0.037 | 0.051 | -0.047 |
|  | (0.103)*** | (0.018) | (0.019)* | (0.024) | (0.034) | (0.027)* |
| Mother has any education | 0.087 | 0.027 | -0.007 | 0.004 | 0.036 | -0.010 |
|  | (0.063) | (0.011)** | (0.012) | (0.015) | (0.021)* | (0.017) |
| Father has any education | -0.013 | 0.022 | 0.012 | 0.009 | -0.027 | -0.005 |
|  | (0.058) | (0.010)** | (0.011) | (0.013) | (0.019) | (0.015) |
| Wealth index in top 60% | 0.198 | 0.003 | 0.012 | -0.010 | 0.004 | 0.011 |
|  | (0.055)*** | (0.010) | (0.010) | (0.013) | (0.018) | (0.014) |
| Surface water not used for drinking | 0.074 | 0.004 | 0.036 | 0.040 | 0.022 | 0.013 |
|  | (0.062) | (0.011) | (0.012)*** | (0.014)*** | (0.020) | (0.016) |
| Any sanitation other than open defecation | 0.080 | -0.010 | 0.014 | 0.003 | 0.034 | 0.081 |
|  | (0.056) | (0.010) | (0.011) | (0.013) | (0.019)* | (0.015)*** |
| Birth order | 0.016 | 0.001 | 0.006 | -0.007 | 0.001 | -0.003 |
|  | (0.017) | (0.003) | (0.003)* | (0.004)* | (0.005) | (0.004) |
| Birth interval > 24 months | 0.157 | 0.030 | -0.016 | -0.026 | -0.025 | 0.010 |
|  | (0.078)** | (0.013)** | (0.015) | (0.018) | (0.025) | (0.020) |
| Mother taller than 150 cm | 0.373 | -0.003 | 0.017 | 0.030 | -0.006 | -0.008 |
|  | (0.074)*** | (0.013) | (0.014) | (0.017)* | (0.024) | (0.019) |
| Mother’s age at birth (in years) | -0.003 | -0.001 | 0.002 | 0.001 | -0.001 | 0.004 |
|  | (0.006) | (0.001) | (0.001) | (0.002) | (0.002) | (0.002)** |
| Have livestock | 0.013 | 0.019 | 0.001 | 0.004 | 0.021 | 0.019 |
|  | (0.090) | (0.016) | (0.017) | (0.021) | (0.029) | (0.023) |
| Urban | -0.015 | 0.042 | -0.053 | 0.031 | 0.126 | 0.013 |
|  | (0.112) | (0.019)** | (0.021)** | (0.026) | (0.036)*** | (0.028) |
| Age - 6-11 months | -0.776 | -0.470 | 0.028 | -0.150 | NA | NA |
|  | (0.068)*** | (0.012)*** | (0.013)** | (0.016)*** |  |  |
| Age - 12-17 months | -1.357 | -0.433 | -0.001 | -0.140 | NA | NA |
|  | (0.069)*** | (0.012)*** | (0.013) | (0.016)*** |  |  |
| Age - 18-23 months | -1.898 | -0.429 | -0.006 | -0.116 | 0.024 | 0.013 |
|  | (0.074)*** | (0.013)*** | (0.014) | (0.017)*** | (0.017) | (0.013) |
| Male | -0.225 | -0.020 | -0.000 | -0.033 | 0.008 | 0.024 |
|  | (0.050)*** | (0.009)** | (0.009) | (0.012)*** | (0.016) | (0.013)* |
| Year=2011 | 0.120 | 0.008 | 0.040 | 0.053 | -0.015 | 0.092 |
|  | (0.056)** | (0.010) | (0.011)*** | (0.013)*** | (0.018) | (0.015)*** |
| Constant | -1.217 | 0.437 | 0.032 | 0.803 | 0.392 | -0.189 |
|  | (0.214)*** | (0.037)*** | (0.040) | (0.049)*** | (0.068)*** | (0.054)*** |
| *R*^2^ | 0.19 | 0.31 | 0.13 | 0.05 | 0.15 | 0.07 |
| *N* | 4,993 | 4,993 | 4,993 | 4,993 | 2,356 | 2,356 |

HAZ = height-for-age z-score. All dependent variables are binary indicators defined as in Table 1. SE = Robust standard errors; * p<0.1; ** p<0.05; *** p<0.01. NA = not available. For the three (two) indicators measured for children aged 0-23 (12-23) months, 0-5 (12-17) months is reference age category. Mother had 0 skilled ANC visits is reference category. Regressions include region indicators.

Table A6. Least squares regressions of indicators of HAZ and proximal determinants of nutritional status of children aged 0-23 and 12-23 months from Ghana

|  | **Children aged 0-23 months** | | | | **Children aged 12-23 months** | |
| --- | --- | --- | --- | --- | --- | --- |
|  | **Recommended age-appropriate feeding** | **Mother received iron supplements** | | **No symptoms of diarrhea** | **Full immunization** | **Deworming medication** |
|  | *Beta (SE)* | *Beta (SE)* | | *Beta (SE)* | *Beta (SE)* | *Beta (SE)* |
| Mother had 1-3 skilled ANC visits | -0.001 | 0.600 | 0.014 | | 0.305 | 0.018 |
|  | (0.058) | (0.036)*** | (0.048) | | (0.079)*** | (0.089) |
| Mother had 4+ skilled ANC visits | 0.012 | 0.656 | -0.009 | | 0.303 | 0.018 |
|  | (0.054) | (0.033)*** | (0.045) | | (0.074)*** | (0.082) |
| Delivered by skilled birth attendant | 0.053 | 0.024 | 0.047 | | 0.045 | 0.019 |
|  | (0.028)* | (0.017) | (0.023)** | | (0.035) | (0.039) |
| Mother has any education | 0.046 | 0.011 | -0.034 | | 0.023 | 0.053 |
|  | (0.030) | (0.018) | (0.025) | | (0.037) | (0.041) |
| Father has any education | 0.034 | 0.000 | 0.079 | | 0.031 | 0.049 |
|  | (0.033) | (0.020) | (0.028)*** | | (0.042) | (0.047) |
| Wealth index in top 60% | 0.018 | -0.017 | 0.027 | | 0.047 | 0.045 |
|  | (0.029) | (0.018) | (0.024) | | (0.037) | (0.042) |
| Surface water not used for drinking | -0.055 | 0.002 | -0.013 | | 0.037 | 0.038 |
|  | (0.037) | (0.022) | (0.030) | | (0.048) | (0.054) |
| Any sanitation other than open defecation | 0.030 | -0.003 | 0.032 | | 0.010 | 0.007 |
|  | (0.032) | (0.019) | (0.026) | | (0.042) | (0.047) |
| Birth order | -0.020 | 0.003 | -0.004 | | -0.021 | 0.007 |
|  | (0.008)** | (0.005) | (0.007) | | (0.010)** | (0.011) |
| Birth interval > 24 months | -0.066 | -0.009 | 0.026 | | -0.022 | 0.012 |
|  | (0.038)* | (0.023) | (0.032) | | (0.050) | (0.056) |
| Mother taller than 150 cm | 0.083 | 0.013 | -0.027 | | 0.038 | -0.077 |
|  | (0.046)* | (0.028) | (0.038) | | (0.059) | (0.065) |
| Mother’s age at birth (in years) | 0.006 | -0.000 | 0.001 | | 0.009 | -0.000 |
|  | (0.002)*** | (0.002) | (0.002) | | (0.003)*** | (0.003) |
| Have livestock | -0.014 | 0.016 | 0.048 | | -0.006 | -0.018 |
|  | (0.025) | (0.015) | (0.020)** | | (0.030) | (0.034) |
| Urban | -0.002 | -0.006 | -0.002 | | -0.115 | -0.008 |
|  | (0.028) | (0.017) | (0.023) | | (0.036)*** | (0.041) |
| Age - 6-11 months | -0.381 | -0.051 | -0.125 | | NA | NA |
|  | (0.030)*** | (0.018)*** | (0.025)*** | |  |  |
| Age - 12-17 months | -0.161 | -0.021 | -0.158 | | NA | NA |
|  | (0.030)*** | (0.018) | (0.025)*** | |  |  |
| Age - 18-23 months | -0.126 | -0.047 | -0.183 | | 0.037 | 0.090 |
|  | (0.031)*** | (0.019)** | (0.026)*** | | (0.027) | (0.030)*** |
| Male | -0.005 | -0.015 | 0.002 | | 0.006 | -0.003 |
|  | (0.021) | (0.013) | (0.018) | | (0.027) | (0.030) |
| Wet season | 0.005 | -0.062 | -0.044 | | 0.015 | -0.042 |
|  | (0.022) | (0.014)*** | (0.019)** | | (0.028) | (0.031) |
| Year=2014 | -0.174 | 0.018 | 0.118 | | -0.063 | -0.092 |
|  | (0.022)*** | (0.014) | (0.018)*** | | (0.028)** | (0.032)*** |
| Constant | 0.447 | 0.306 | 0.763 | | 0.194 | 0.414 |
|  | (0.106)*** | (0.065)*** | (0.088)*** | | (0.136) | (0.152)*** |
| *R*^2^ | 0.15 | 0.23 | 0.10 | | 0.10 | 0.16 |
| *N* | 1,845 | 1,845 | 1,845 | | 903 | 903 |

As Table A2.

Table A7. Least squares regressions of indicators of HAZ and proximal determinants of nutritional status of children aged 0-23 and 12-23 months from Kenya

|  | **Children aged 0-23 months** | | | | | **Children aged 12-23 months** | |
| --- | --- | --- | --- | --- | --- | --- | --- |
|  | **HAZ** | **Recommended age-appropriate feeding** | **Mother received iron supplements** | **No symptoms of diarrhea** | | **Full immunization** | **Deworming medication** |
|  | *Beta (SE)* | *Beta (SE)* | *Beta (SE)* | *Beta (SE)* | | *Beta (SE)* | *Beta (SE)* |
| Mother had 1-3 skilled ANC visits | -0.234 | 0.043 | 0.527 | -0.007 | 0.155 | | 0.044 |
|  | (0.109)** | (0.031) | (0.030)*** | (0.029) | (0.046)*** | | (0.050) |
| Mother had 4+ skilled ANC visits | -0.128 | 0.018 | 0.621 | -0.006 | 0.148 | | 0.105 |
|  | (0.111) | (0.031) | (0.030)*** | (0.030) | (0.047)*** | | (0.051)** |
| Delivered by skilled birth attendant | 0.206 | 0.050 | 0.040 | 0.001 | 0.021 | | 0.050 |
|  | (0.055)*** | (0.016)*** | (0.015)*** | (0.015) | (0.022) | | (0.024)** |
| Mother has any education | -0.064 | 0.026 | -0.001 | 0.053 | 0.000 | | 0.098 |
|  | (0.096) | (0.027) | (0.026) | (0.026)** | (0.038) | | (0.042)** |
| Father has any education | -0.072 | 0.036 | -0.008 | -0.073 | 0.101 | | 0.044 |
|  | (0.103) | (0.029) | (0.028) | (0.028)*** | (0.041)** | | (0.044) |
| Wealth index in top 60% | 0.224 | 0.057 | 0.003 | -0.009 | -0.018 | | 0.011 |
|  | (0.061)*** | (0.017)*** | (0.017) | (0.017) | (0.024) | | (0.027) |
| Surface water not used for drinking | 0.077 | 0.008 | 0.006 | 0.038 | -0.009 | | -0.024 |
|  | (0.059) | (0.016) | (0.016) | (0.016)** | (0.023) | | (0.026) |
| Any sanitation other than open defecation | -0.110 | 0.037 | -0.013 | 0.071 | 0.065 | | 0.059 |
|  | (0.074) | (0.021)* | (0.020) | (0.020)*** | (0.030)** | | (0.032)* |
| Birth order | -0.037 | 0.010 | 0.038 | -0.029 | 0.022 | | 0.035 |
|  | (0.017)** | (0.016) | (0.016)** | (0.016)* | (0.023) | | (0.025) |
| Birth interval > 24 months | 0.147 | -0.014 | -0.011 | -0.015 | -0.047 | | -0.007 |
|  | (0.068)** | (0.005)*** | (0.005)** | (0.005)*** | (0.007)*** | | (0.007) |
| Mother taller than 150 cm | 0.638 | -0.050 | 0.010 | 0.008 | 0.041 | | -0.072 |
|  | (0.095)*** | (0.019)*** | (0.018) | (0.018) | (0.027) | | (0.029)** |
| Mother’s age at birth (in years) | 0.015 | 0.033 | -0.054 | 0.060 | -0.112 | | -0.046 |
|  | (0.006)*** | (0.027) | (0.026)** | (0.026)** | (0.037)*** | | (0.040) |
| Having livestock | 0.003 | 0.005 | 0.001 | 0.008 | 0.008 | | -0.003 |
|  | (0.057) | (0.002)*** | (0.002) | (0.002)*** | (0.002)*** | | (0.002) |
| Urban | 0.229 | 0.062 | 0.031 | -0.033 | -0.091 | | 0.008 |
|  | (0.070)*** | (0.020)*** | (0.019) | (0.019)* | (0.028)*** | | (0.031) |
| Age - 6-11 months | -0.394 | -0.353 | -0.026 | -0.146 | NA | | NA |
|  | (0.064)*** | (0.018)*** | (0.018) | (0.017)*** |  | |  |
| Age - 12-17 months | -1.116 | -0.203 | -0.027 | -0.158 | NA | | NA |
|  | (0.065)*** | (0.018)*** | (0.018) | (0.017)*** |  | |  |
| Age - 18-23 months | -1.263 | -0.166 | -0.050 | -0.136 | 0.023 | | 0.084 |
|  | (0.066)*** | (0.019)*** | (0.018)*** | (0.018)*** | (0.018) | | (0.020)*** |
| Male | -0.184 | -0.010 | 0.005 | 0.005 | 0.010 | | 0.026 |
|  | (0.045)*** | (0.013) | (0.012) | (0.012) | (0.018) | | (0.019) |
| Year=2014 | 0.079 | 0.073 | -0.043 | 0.027 | 0.043 | | 0.030 |
|  | (0.049) | (0.014)*** | (0.013)*** | (0.013)** | (0.019)** | | (0.021) |
| Constant | -1.280 | 0.170 | 0.305 | 0.532 | 0.500 | | 0.194 |
|  | (0.210)*** | (0.059)*** | (0.057)*** | (0.057)*** | (0.086)*** | | (0.094)** |
| *R*^2^ | 0.14 | 0.14 | 0.15 | 0.05 | 0.08 | | 0.07 |
| *N* | 4,795 | 4,795 | 4,795 | 4,795 | 2,368 | | 2,368 |

As Table A2.

Table A8. Least squares regressions of indicators of HAZ and proximal determinants of nutritional status of children aged 0-23 and 12-23 months from Liberia

|  | **Children aged 0-23 months** | | | | **Children aged 12-23 months** | |
| --- | --- | --- | --- | --- | --- | --- |
|  | **HAZ** | **Recommended age-appropriate feeding** | **Mother received iron supplements** | **No symptoms of diarrhea** | **Full immunization** | **Deworming medication** |
|  | *Beta (SE)* | *Beta (SE)* | *Beta (SE)* | *Beta (SE)* | *Beta (SE)* | *Beta (SE)* |
| Mother had 1-3 skilled ANC visits | -0.098 | 0.115 | 0.263 | 0.101 | 0.078 | 0.004 |
|  | (0.146) | (0.033)*** | (0.022)*** | (0.035)*** | (0.064) | (0.066) |
| Mother had 4+ skilled ANC visits | -0.061 | 0.116 | 0.328 | 0.123 | 0.200 | 0.103 |
|  | (0.124) | (0.028)*** | (0.019)*** | (0.030)*** | (0.052)*** | (0.053)* |
| Delivered by skilled birth attendant | 0.090 | 0.036 | 0.003 | -0.025 | 0.122 | -0.029 |
|  | (0.091) | (0.020)* | (0.014) | (0.022) | (0.037)*** | (0.038) |
| Mother has any education | -0.016 | 0.017 | 0.022 | 0.045 | -0.001 | 0.027 |
|  | (0.087) | (0.020) | (0.013)* | (0.021)** | (0.035) | (0.036) |
| Father has any education | 0.183 | -0.029 | -0.010 | -0.038 | -0.003 | 0.001 |
|  | (0.096)* | (0.022) | (0.015) | (0.023) | (0.038) | (0.039) |
| Wealth index in top 60% | 0.027 | 0.025 | 0.035 | -0.022 | -0.015 | 0.077 |
|  | (0.102) | (0.023) | (0.016)** | (0.025) | (0.044) | (0.045)* |
| Surface water not used for drinking | 0.181 | 0.024 | 0.079 | 0.014 | 0.080 | 0.078 |
|  | (0.117) | (0.026) | (0.018)*** | (0.028) | (0.049) | (0.050) |
| Any sanitation other than open defecation | 0.107 | 0.038 | -0.011 | -0.046 | 0.056 | -0.001 |
|  | (0.088) | (0.020)* | (0.014) | (0.021)** | (0.036) | (0.037) |
| Birth order | -0.016 | -0.001 | -0.010 | -0.020 | -0.014 | -0.020 |
|  | (0.027) | (0.006) | (0.004)** | (0.007)*** | (0.012) | (0.012) |
| Birth interval > 24 months | 0.145 | -0.034 | -0.021 | 0.005 | -0.001 | -0.077 |
|  | (0.130) | (0.029) | (0.020) | (0.031) | (0.056) | (0.057) |
| Mother taller than 150 cm | 0.282 | -0.017 | 0.039 | 0.063 | 0.064 | -0.018 |
|  | (0.118)** | (0.027) | (0.018)** | (0.028)** | (0.046) | (0.048) |
| Mother’s age at birth (in years) | 0.016 | 0.001 | 0.001 | 0.007 | -0.000 | 0.002 |
|  | (0.009)* | (0.002) | (0.001) | (0.002)*** | (0.004) | (0.004) |
| Have livestock | -0.171 | -0.024 | 0.022 | -0.030 | 0.027 | 0.044 |
|  | (0.081)** | (0.018) | (0.012)* | (0.019) | (0.033) | (0.034) |
| Urban | -0.005 | -0.035 | -0.019 | 0.040 | -0.028 | 0.029 |
|  | (0.115) | (0.026) | (0.018) | (0.028) | (0.048) | (0.049) |
| Age - 6-11 months | -0.373 | -0.299 | -0.015 | -0.236 | NA | NA |
|  | (0.106)*** | (0.024)*** | (0.016) | (0.025)*** |  |  |
| Age - 12-17 months | -1.135 | -0.220 | 0.004 | -0.210 | NA | NA |
|  | (0.112)*** | (0.025)*** | (0.017) | (0.027)*** |  |  |
| Age - 18-23 months | -1.529 | -0.177 | 0.001 | -0.211 | 0.004 | 0.043 |
|  | (0.112)*** | (0.025)*** | (0.017) | (0.027)*** | (0.031) | (0.032) |
| Male | -0.222 | -0.022 | 0.031 | -0.035 | -0.041 | -0.039 |
|  | (0.077)*** | (0.017) | (0.012)*** | (0.018)* | (0.032) | (0.033) |
| Wet season | 0.158 | 0.039 | -0.029 | -0.080 | -0.007 | 0.004 |
|  | (0.161) | (0.036) | (0.025) | (0.039)** | (0.065) | (0.066) |
| Year=2013 | 0.115 | -0.100 | 0.051 | 0.035 | 0.040 | 0.075 |
|  | (0.155) | (0.035)*** | (0.024)** | (0.037) | (0.062) | (0.063) |
| Constant | -1.114 | 0.312 | 0.520 | 0.831 | 0.236 | 0.323 |
|  | (0.292)*** | (0.066)*** | (0.045)*** | (0.070)*** | (0.121)* | (0.124)*** |
| *R*^2^ | 0.13 | 0.10 | 0.24 | 0.09 | 0.10 | 0.08 |
| *N* | 2,084 | 2,084 | 2,084 | 2,084 | 935 | 935 |

As Table A2.

Table A9. Least squares regressions of indicators of HAZ and proximal determinants of nutritional status of children aged 0-23 and 12-23 months from Namibia

|  | **Children aged 0-23 months** | | | | | **Children aged 12-23 months** | |
| --- | --- | --- | --- | --- | --- | --- | --- |
|  | **HAZ** | **Recommended age-appropriate feeding** | **Mother received iron supplements** | **No symptoms of diarrhea** | | **Full immunization** | **Deworming medication** |
|  | *Beta (SE)* | *Beta (SE)* | *Beta (SE)* | *Beta (SE)* | | *Beta (SE)* | *Beta (SE)* |
| Mother had 1-3 skilled ANC visits | 0.607 | -0.075 | 0.603 | 0.049 | -0.072 | | -0.042 |
|  | (0.221)*** | (0.059) | (0.049)*** | (0.058) | (0.093) | | (0.077) |
| Mother had 4+ skilled ANC visits | 0.812 | -0.078 | 0.692 | 0.043 | -0.019 | | -0.010 |
|  | (0.207)*** | (0.055) | (0.046)*** | (0.055) | (0.086) | | (0.071) |
| Delivered by skilled birth attendant | -0.117 | -0.013 | 0.014 | -0.081 | 0.157 | | 0.132 |
|  | (0.148) | (0.040) | (0.033) | (0.039)** | (0.063)** | | (0.052)** |
| Mother has any education | 0.090 | 0.050 | 0.027 | 0.066 | 0.189 | | -0.005 |
|  | (0.172) | (0.046) | (0.038) | (0.045) | (0.068)*** | | (0.057) |
| Father has any education | -0.207 | -0.041 | -0.022 | -0.044 | 0.004 | | -0.098 |
|  | (0.150) | (0.040) | (0.033) | (0.040) | (0.059) | | (0.049)** |
| Wealth index in top 60% | 0.216 | 0.056 | 0.023 | 0.009 | 0.023 | | -0.019 |
|  | (0.148) | (0.040) | (0.033) | (0.039) | (0.063) | | (0.052) |
| Surface water not used for drinking | 0.587 | 0.066 | 0.017 | 0.110 | -0.052 | | 0.004 |
|  | (0.199)*** | (0.054) | (0.044) | (0.053)** | (0.082) | | (0.068) |
| Any sanitation other than open defecation | 0.208 | 0.106 | -0.019 | 0.000 | 0.036 | | 0.091 |
|  | (0.149) | (0.040)*** | (0.033) | (0.039) | (0.063) | | (0.052)* |
| Birth order | -0.080 | -0.008 | 0.009 | -0.026 | -0.021 | | 0.014 |
|  | (0.037)** | (0.010) | (0.008) | (0.010)*** | (0.016) | | (0.014) |
| Birth interval > 24 months | -0.163 | -0.044 | 0.024 | 0.064 | -0.008 | | 0.008 |
|  | (0.164) | (0.044) | (0.036) | (0.043) | (0.067) | | (0.056) |
| Mother taller than 150 cm | 0.625 | 0.076 | 0.032 | -0.036 | -0.073 | | 0.068 |
|  | (0.225)*** | (0.060) | (0.050) | (0.059) | (0.093) | | (0.077) |
| Mother’s age at birth (in years) | 0.021 | -0.002 | -0.005 | 0.013 | 0.005 | | -0.007 |
|  | (0.010)** | (0.003) | (0.002)** | (0.003)*** | (0.005) | | (0.004)* |
| Having livestock | 0.153 | -0.032 | -0.040 | -0.046 | -0.027 | | -0.043 |
|  | (0.118) | (0.032) | (0.026) | (0.031) | (0.052) | | (0.043) |
| Urban | 0.097 | 0.089 | -0.017 | 0.004 | -0.044 | | -0.054 |
|  | (0.152) | (0.041)** | (0.034) | (0.040) | (0.067) | | (0.056) |
| Age - 6-11 months | 0.093 | -0.071 | -0.007 | -0.123 | NA | | NA |
|  | (0.131) | (0.035)** | (0.029) | (0.035)*** |  | |  |
| Age - 12-17 months | -0.692 | -0.009 | -0.019 | -0.193 | NA | | NA |
|  | (0.135)*** | (0.036) | (0.030) | (0.036)*** |  | |  |
| Age - 18-23 months | -1.114 | 0.120 | -0.050 | -0.121 | 0.070 | | 0.040 |
|  | (0.141)*** | (0.038)*** | (0.031) | (0.037)*** | (0.040)* | | (0.034) |
| Male | -0.308 | -0.002 | -0.007 | -0.035 | -0.063 | | -0.052 |
|  | (0.096)*** | (0.026) | (0.021) | (0.025) | (0.040) | | (0.033) |
| Year=2013 | 0.518 | 0.120 | 0.045 | -0.137 | 0.019 | | 0.396 |
|  | (0.113)*** | (0.030)*** | (0.025)* | (0.030)*** | (0.048) | | (0.040)*** |
| Constant | -2.680 | 0.236 | 0.164 | 0.502 | 0.562 | | 0.307 |
|  | (0.467)*** | (0.125)* | (0.103) | (0.124)*** | (0.193)*** | | (0.160)* |
| *R*^2^ | 0.19 | 0.16 | 0.26 | 0.12 | 0.17 | | 0.30 |
| *N* | 1,015 | 1,015 | 1,015 | 1,015 | 480 | | 480 |

As Table A2.

Table A10. Least squares regressions of indicators of HAZ and proximal determinants of nutritional status of children aged 0-23 and 12-23 months from Niger

|  | **Children aged 0-23 months** | | | | **Children aged 12-23 months** |
| --- | --- | --- | --- | --- | --- |
|  | **HAZ** | **Recommended age-appropriate feeding** | **Mother received iron supplements** | **No symptoms of diarrhea** | **Full immunization** |
|  | *Beta (SE)* | *Beta (SE)* | *Beta (SE)* | *Beta (SE)* | *Beta (SE)* |
| Mother had 1-3 skilled ANC visits | -0.098 | 0.041 | 0.730 | -0.002 | 0.237 |
|  | (0.088) | (0.016)*** | (0.015)*** | (0.022) | (0.032)*** |
| Mother had 4+ skilled ANC visits | -0.058 | 0.056 | 0.784 | -0.022 | 0.269 |
|  | (0.099) | (0.018)*** | (0.017)*** | (0.025) | (0.036)*** |
| Delivered by skilled birth attendant | -0.064 | 0.019 | 0.025 | -0.000 | 0.144 |
|  | (0.085) | (0.015) | (0.014)* | (0.022) | (0.030)*** |
| Mother has any education | 0.088 | 0.031 | -0.002 | 0.042 | 0.082 |
|  | (0.100) | (0.018)* | (0.017) | (0.025)* | (0.036)** |
| Father has any education | 0.037 | -0.002 | 0.056 | 0.005 | 0.034 |
|  | (0.090) | (0.016) | (0.015)*** | (0.023) | (0.033) |
| Wealth index in top 60% | -0.011 | -0.003 | -0.001 | -0.028 | 0.038 |
|  | (0.077) | (0.014) | (0.013) | (0.020) | (0.028) |
| Surface water not used for drinking | 0.459 | -0.102 | 0.007 | 0.078 | 0.034 |
|  | (0.264)* | (0.048)** | (0.045) | (0.068) | (0.105) |
| Any sanitation other than open defecation | 0.325 | 0.038 | 0.023 | 0.004 | 0.098 |
|  | (0.099)*** | (0.018)** | (0.017) | (0.025) | (0.036)*** |
| Birth order | -0.053 | -0.007 | -0.000 | 0.001 | -0.010 |
|  | (0.022)** | (0.004)* | (0.004) | (0.006) | (0.008) |
| Birth interval > 24 months | 0.101 | 0.029 | -0.003 | -0.018 | 0.001 |
|  | (0.092) | (0.017)* | (0.016) | (0.023) | (0.032) |
| Mother taller than 150 cm | 0.262 | 0.044 | 0.031 | 0.013 | -0.025 |
|  | (0.181) | (0.033) | (0.031) | (0.046) | (0.063) |
| Mother’s age at birth (in years) | 0.027 | 0.001 | 0.000 | 0.002 | 0.002 |
|  | (0.009)*** | (0.002) | (0.001) | (0.002) | (0.003) |
| Urban | 0.338 | 0.098 | -0.066 | 0.069 | -0.062 |
|  | (0.142)** | (0.026)*** | (0.024)*** | (0.036)* | (0.052) |
| Age - 6-11 months | -0.521 | -0.192 | 0.020 | -0.153 | NA |
|  | (0.096)*** | (0.017)*** | (0.016) | (0.025)*** |  |
| Age - 12-17 months | -1.182 | -0.120 | 0.022 | -0.151 | NA |
|  | (0.096)*** | (0.017)*** | (0.016) | (0.025)*** |  |
| Age - 18-23 months | -1.868 | -0.108 | 0.055 | -0.096 | 0.106 |
|  | (0.104)*** | (0.019)*** | (0.018)*** | (0.027)*** | (0.024)*** |
| Male | -0.265 | -0.001 | -0.012 | -0.002 | -0.058 |
|  | (0.064)*** | (0.012) | (0.011) | (0.016) | (0.023)** |
| Wet season | -0.059 | -0.006 | -0.010 | 0.062 | 0.018 |
|  | (0.076) | (0.014) | (0.013) | (0.019)*** | (0.028) |
| Year=2012 | 0.525 | -0.053 | 0.083 | 0.044 | 0.081 |
|  | (0.083)*** | (0.015)*** | (0.014)*** | (0.021)** | (0.028)*** |
| Constant | -1.872 | 0.189 | -0.140 | 0.640 | 0.148 |
|  | (0.473)*** | (0.086)** | (0.080)* | (0.121)*** | (0.173) |
| *R*^2^ | 0.19 | 0.10 | 0.61 | 0.04 | 0.21 |
| *N* | 2,875 | 2,875 | 2,875 | 2,875 | 1,459 |

As Table A2.

Table A11. Least squares regressions of indicators of HAZ and proximal determinants of nutritional status of children aged 0-23 and 12-23 months from Rwanda

|  |  | | **Children aged 0-23 months** | | **Children aged 12-23 months** | |
| --- | --- | --- | --- | --- | --- | --- |
|  | **HAZ** | **Recommended age-appropriate feeding** | **Mother received iron supplements** | **No symptoms of diarrhea** | **Full immunization** | **Deworming medication** |
|  | *Beta (SE)* | *Beta (SE)* | *Beta (SE)* | *Beta (SE)* | *Beta (SE)* | *Beta (SE)* |
| Mother had 1-3 skilled ANC visits | 0.213 | 0.060 | 0.534 | 0.106 | 0.264 | 0.092 |
|  | (0.268) | (0.072) | (0.073)*** | (0.071) | (0.071)*** | (0.098) |
| Mother had 4+ skilled ANC visits | 0.401 | 0.059 | 0.586 | 0.096 | 0.266 | 0.095 |
|  | (0.270) | (0.073) | (0.074)*** | (0.072) | (0.071)*** | (0.099) |
| Delivered by skilled birth attendant | 0.232 | -0.003 | 0.115 | -0.015 | 0.042 | 0.030 |
|  | (0.087)*** | (0.023) | (0.024)*** | (0.023) | (0.021)** | (0.029) |
| Mother has any education | 0.131 | 0.025 | 0.028 | -0.002 | 0.015 | 0.076 |
|  | (0.083) | (0.022) | (0.023) | (0.022) | (0.021) | (0.029)*** |
| Father has any education | 0.064 | 0.003 | 0.032 | 0.004 | 0.102 | 0.046 |
|  | (0.079) | (0.021) | (0.022) | (0.021) | (0.020)*** | (0.028) |
| Wealth index in top 60% | 0.255 | 0.075 | 0.025 | -0.005 | 0.003 | -0.016 |
|  | (0.065)*** | (0.017)*** | (0.018) | (0.017) | (0.017) | (0.023) |
| Surface water not used for drinking | 0.030 | 0.057 | -0.057 | -0.010 | -0.008 | -0.013 |
|  | (0.097) | (0.026)** | (0.026)** | (0.026) | (0.024) | (0.033) |
| Any sanitation other than open defecation | 0.065 | -0.006 | -0.003 | -0.003 | -0.013 | -0.005 |
|  | (0.192) | (0.006) | (0.006) | (0.006) | (0.006)** | (0.008) |
| Birth order | -0.037 | 0.011 | -0.026 | 0.003 | -0.039 | -0.040 |
|  | (0.022)* | (0.023) | (0.023) | (0.023) | (0.022)* | (0.030) |
| Birth interval > 24 months | 0.123 | 0.005 | 0.026 | 0.007 | 0.033 | 0.045 |
|  | (0.085) | (0.024) | (0.024) | (0.023) | (0.023) | (0.032) |
| Mother taller than 150 cm | 0.503 | 0.002 | 0.002 | 0.002 | 0.006 | 0.003 |
|  | (0.088)*** | (0.002) | (0.002) | (0.002) | (0.002)*** | (0.003) |
| Mother’s age at birth (in years) | 0.013 | -0.011 | -0.031 | 0.149 | 0.089 | 0.055 |
|  | (0.008)* | (0.052) | (0.053) | (0.051)*** | (0.051)* | (0.071) |
| Having livestock | 0.062 | -0.001 | 0.022 | 0.040 | 0.015 | 0.022 |
|  | (0.060) | (0.016) | (0.016) | (0.016)** | (0.016) | (0.022) |
| Urban | 0.238 | 0.052 | -0.009 | -0.011 | 0.011 | -0.003 |
|  | (0.104)** | (0.028)* | (0.029) | (0.028) | (0.027) | (0.038) |
| Age - 6-11 months | -0.409 | -0.710 | 0.053 | -0.128 | NA | NA |
|  | (0.080)*** | (0.022)*** | (0.022)** | (0.021)*** |  |  |
| Age - 12-17 months | -1.146 | -0.609 | 0.008 | -0.197 | NA | NA |
|  | (0.082)*** | (0.022)*** | (0.022) | (0.022)*** |  |  |
| Age - 18-23 months | -1.411 | -0.613 | 0.007 | -0.171 | -0.003 | 0.158 |
|  | (0.083)*** | (0.022)*** | (0.023) | (0.022)*** | (0.015) | (0.020)*** |
| Male | -0.379 | -0.023 | 0.008 | -0.050 | 0.003 | 0.025 |
|  | (0.057)*** | (0.015) | (0.016) | (0.015)*** | (0.015) | (0.020) |
| Wet season | -0.337 | -0.054 | -0.019 | -0.051 | 0.159 | 0.198 |
|  | (0.224) | (0.060) | (0.061) | (0.060) | (0.060)*** | (0.082)** |
| Year=2014 | -0.008 | 0.014 | -0.014 | 0.019 | 0.009 | -0.013 |
|  | (0.059) | (0.016) | (0.016) | (0.016) | (0.015) | (0.021) |
| Constant | -1.570 | 0.716 | 0.109 | 0.752 | 0.196 | 0.239 |
|  | (0.475)*** | (0.128)*** | (0.130) | (0.127)*** | (0.125) | (0.173) |
| *R*^2^ | 0.19 | 0.35 | 0.07 | 0.05 | 0.08 | 0.07 |
| *N* | 2,603 | 2,603 | 2,603 | 2,603 | 1,284 | 1,284 |

As Table A2.

**
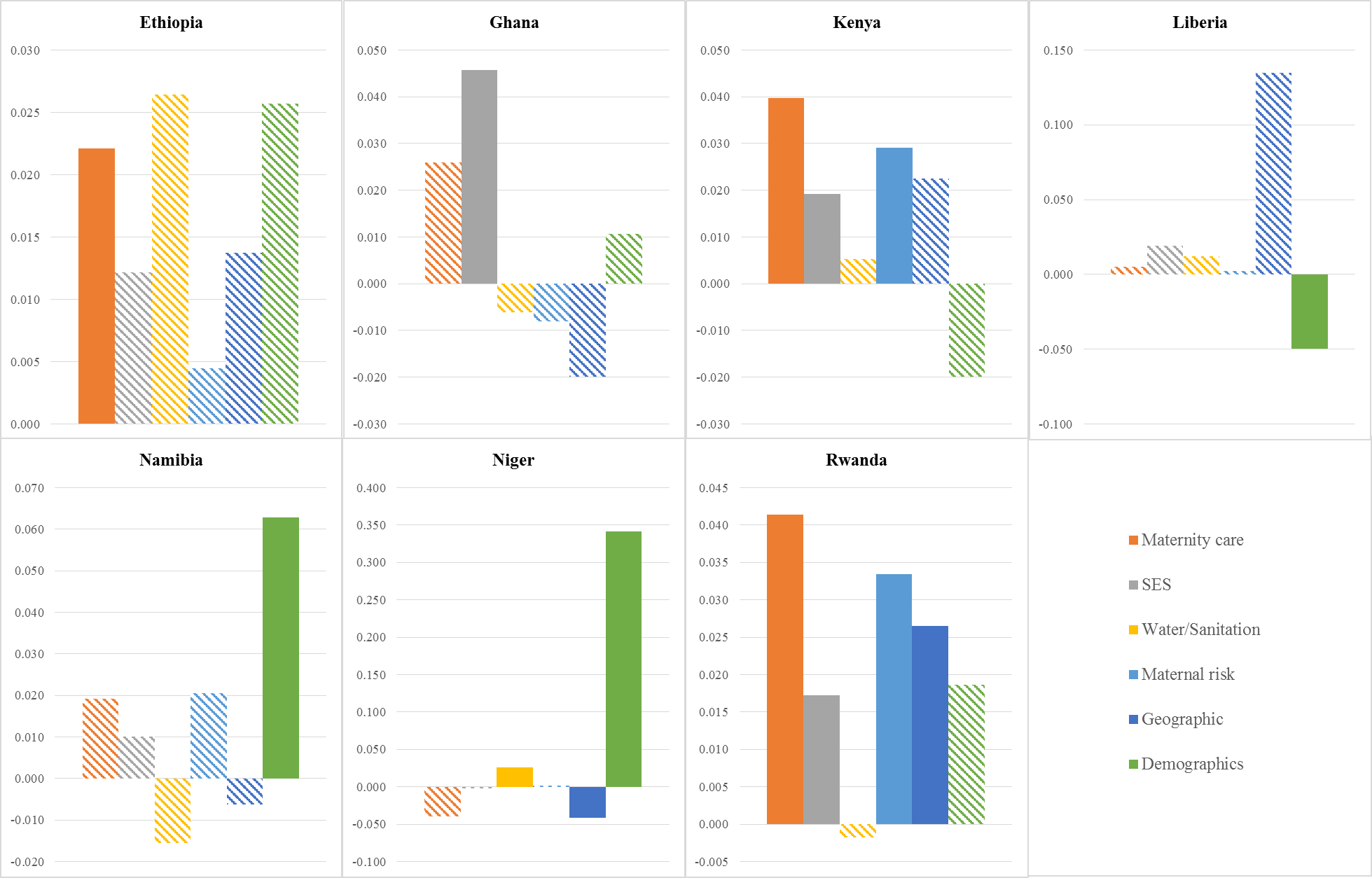
**

Figure A1. Contributions of changes in distal determinants and covariates to changes in mean height-for-age by country, children aged 0-23 months

Significant at 10% level: non-striped shading; Groups of determinants: (1) Maternity care: Mother had 4+ skilled ANC visits, Delivered by skilled birth attendant; (2) Socioeconomic status: Mother has any education, Father has any education, Wealth index in top 60%, Having livestock; (3) Water/Sanitation: Surface water not used for drinking, Any sanitation other than open defecation; (4) Maternal risk: Birth order, Birth interval>24 months, Mother taller than 150 cm, Mother’s age at birth; (5) Geographic characteristics: Urban, Regions; (6) Demographics: Child's age, Child is male.


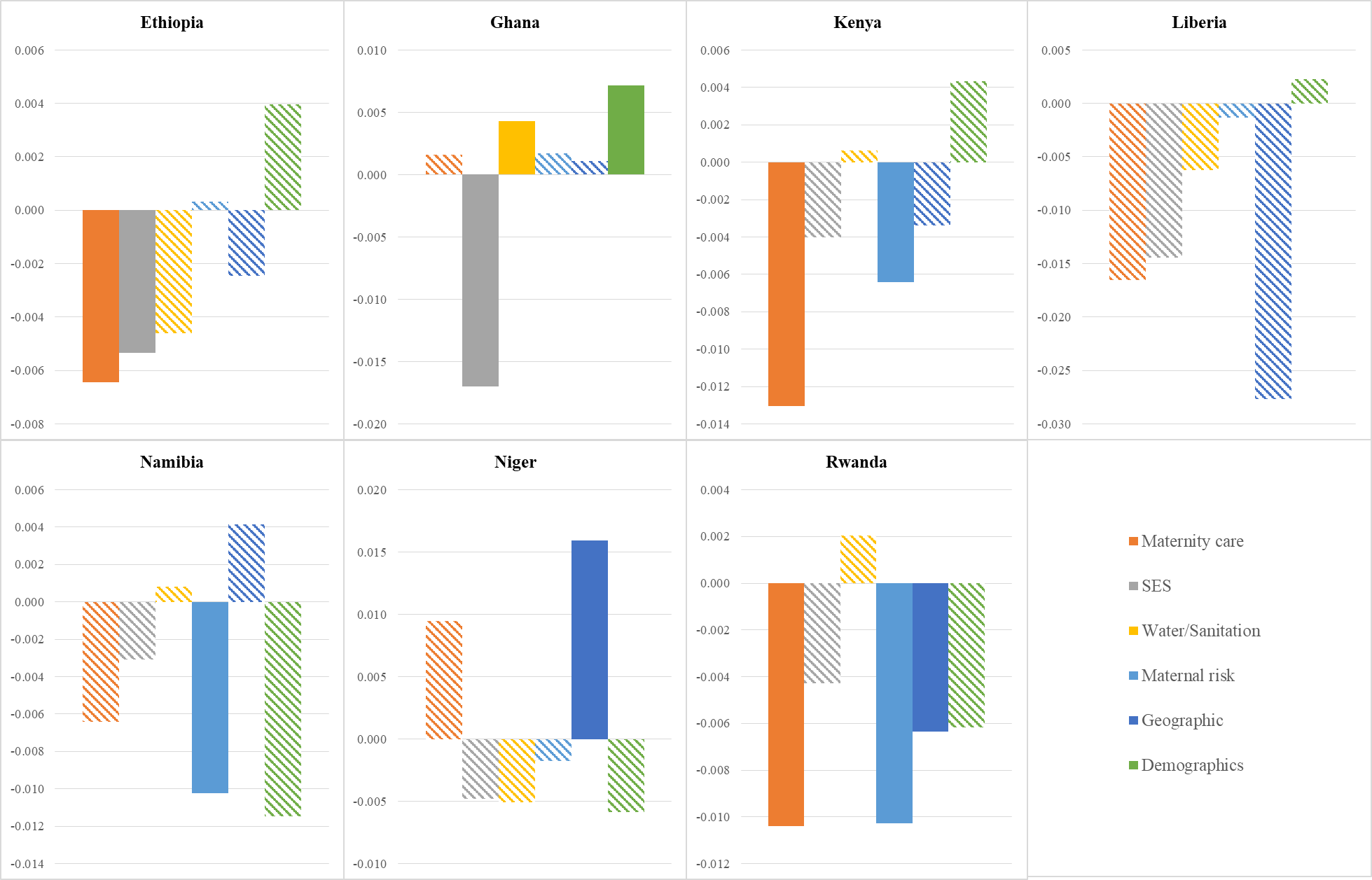


Figure A2. Contributions of changes in distal determinants and covariates to changes in stunting by country, children aged 6-23 months, robustness check

Significant at 10% level: non-striped shading; Groups of determinants: (1) Maternity care: Mother had 4+ skilled ANC visits, Delivered by skilled birth attendant; (2) Socioeconomic status: Mother has any education, Father has any education, Wealth index in top 60%, Having livestock; (3) Water/Sanitation: Surface water not used for drinking, Any sanitation other than open defecation; (4) Maternal risk: Birth order, Birth interval>24 months, Mother taller than 150 cm, Mother’s age at birth; (5) Geographic characteristics: Urban, Regions; (6) Demographics: Child's age, Child is male.
